# Green-Synthesized Silver and Nickel Oxide Nanoparticles as Photostabilizers for PVC Films Under Accelerated UV Irradiation

**DOI:** 10.3390/polym18202512

**Published:** 2026-10-14

**Authors:** Saif Saad Abdulsattar Al-Shaikhli, Khalid Umar, Emad Yousif

**Affiliations:** 1 School of Chemical Sciences, Universiti Sains Malaysia, Gelugor 11800, Penang, Malaysia; 2 Department of Chemistry, College of Science, Al-Nahrain University, Baghdad 10072, Iraq

**Keywords:** green synthesis, UV radiation, photodegradation, PVC, morphology

## Abstract

Poly(vinyl chloride) shows gradual photodegradation by dehydrochlorination and oxidative chain reactions under long-term UV irradiation. Although conventional PVC stabilizers may limit the photodegradation, concerns about toxicity, migration of additives and long-term environmental effects have stimulated the search for new stabilizing solutions. Ag and NiO NPs materials were synthesized utilizing a plant-based green approach and added to PVC/CNNP/GO films at 0.3 and 0.5 wt%. The nanomaterials were characterized using different techniques, such as UV-Vis, FTIR, FESEM, EDX, TEM, and AFM. Accelerated UV-B irradiation assessed photodegradation via carbonyl, polyene, and hydroxyl indices; weight loss; and surface morphology. Nanoparticle-containing films showed less degradation than did blank PVC. The best film, with 0.5 wt% Ag NPs after prolonged irradiation, achieved carbonyl, polyene, and hydroxyl indices of 0.551, 0.545, and 0.591. Weight loss dropped from 2.242% for blank PVC to 1.131% for the Ag-containing film after 300 h, and the NiO NPs improved photostability but provided less protection than did Ag NPs at the same loading. These findings suggest that Ag and NiO NPs might prevent UV-induced PVC breakdown when used as nanomaterial-based photostabilizing additives.

## 1. Introduction

Poly(vinyl chloride) (PVC) is one of the most widely used thermoplastic polymers because of its low cost, good mechanical strength, chemical resistance, and easy processing [1]. It is commonly used in construction, electrical insulation, packaging, medical devices, and outdoor products. Nevertheless, extended exposure to ultraviolet (UV) radiation, oxygen, and heat causes the PVC to become heavily degraded, and consequently, its long-term outdoor endurance deteriorates [2].

PVC photodegradation is generally initiated by UV-induced dehydrochlorination, with hydrogen chloride release and the formation of conjugated polyene sequences [3]. Further oxidation gives rise to carbonyl, hydroxyl, peroxide, and hydroperoxide groups, which speed up chain scission, discoloration, embrittlement, surface cracking, and mechanical loss. The released HCl can also cause autocatalytic degradation, necessitating stabilization for long-term PVC applications [4].

Traditional stabilizers such as organotin compounds, lead salts, Ca/Zn systems, UV absorbers, and hindered amine stabilizers are employed for delaying PVC degradation [5]. Nevertheless, toxicity, environmental concerns, migration from the polymer matrix, and reduced long-term efficiency have all motivated the development of safer and more sustainable options [6]. In this context, nanomaterials have attracted considerable research attention due to their high surface area, optical activity, radical scavenging ability, and strong interaction with polymer matrices [7].

The silver nanoparticles Ag NPs and nickel oxide nanoparticles NiO NPs have become promising materials for PVC photostabilization, as they are considered to work by complementary mechanisms. Ag NPs can absorb and scatter UV radiation through surface plasmon behavior and inhibit radical propagation [8]. NiO NPs are p-type semiconductor materials and have good thermal and chemical stability, which could result in electron transfer, radical trapping, and the reduction of photooxidative reactions [9]. Green synthesis further enhances their ecological importance by employing plant-derived phytochemicals as reducing and stabilizing agents [10].

While silver and nickel oxide nanoparticles have been studied in various polymer systems, direct comparison of green-synthesized silver nanoparticles and nickel oxide nanoparticles as PVC photostabilizers under identical experimental conditions remains limited [11]. Thus, this work intends to synthesize and characterize selected silver and nickel oxide nanoparticles and determine their efficiency for the enhancement of the photostability of the PVC film under accelerated UV light irradiation.

The inorganic nanophotostabilizers Ag and NiO NPs were selected for their complementary optical and surface properties. UV attenuation and radical suppression may be caused by Ag NPs’ strong light–matter interaction and high surface activity. As p-type semiconductor nanoparticles, NiO NPs may aid UV absorption, charge transfer, and radical trapping. In the PVC/CNNP/GO matrix, these effects should slow dehydrochlorination and oxidative chain reactions.

Few studies have directly compared green-synthesized Ag NPs and NiO NPs as photostabilizers in PVC/CNNP/GO systems under accelerated UV-B conditions. Comparing these two nanoparticle forms at low loadings of 0.3 and 0.5 wt% in the same PVC/CNNP/GO matrix using complementary chemical, gravimetric, morphological, and surface studies is novel. Carbonyl, polyene, and hydroxyl indices; weight loss; FESEM morphology; AFM roughness; and elemental mapping were used to evaluate photostability. The addition of Ag or NiO nanomaterials was hypothesized to suppress UV-induced dehydrochlorination and photooxidation, minimize mass loss and surface damage, and provide protection, depending on nanoparticle type and loading. In order to establish the best photostabilization formulation in the measured concentration range, the two nanoparticle systems were examined.

## 2. Materials and Methodology

### 2.1. Material and Instrumentals

All chemicals used in this study, such as PVC, melamine, AgNO_3_, Ni(NO_3_)_2_·6H_2_O, H_2_SO_4_, and HNO_3_, were procured from CDH (India), while THF and absolute ethanol were obtained from Sigma-Aldrich.

FTIR spectra were captured using a Shimadzu Model 8300 (Japan) spectrophotometer in the 4000–400 cm^−1^ range. For surface morphology, a ZEISS Sigma VP field-emission scanning electron microscope was used, and elemental composition was measured with an Oxford Instruments EDX (UK). AFM was performed using a Veeco (USA) AFM, and Fast UV exposure was completed using a QUV weathering tester with UV-B 313 lamps. The samples were palced 25 cm from the radiation source, and the irradiation wavelength was 290–360 nm, with maximum intensity at 313 nm.

### 2.2. Preparation of Carbon Nitride Nanoparticles Hybrid Nanocomposite (CNNPs)

Bulk graphitic carbon nitride (CN) was prepared by direct thermal condensation of melamine under semi-closed conditions. Briefly, 5.0 g of melamine was placed in a covered 20 mL ceramic crucible and heated in a muffle furnace at 600 °C for 2 h using a heating rate of 3 °C min^−1^. After natural cooling to room temperature, the resulting light-yellow agglomerates were collected and ground into a fine powder. Subsequently, carbon nitride nanoparticles (CNNPs) were made from the collected CN powder via disintegration–exfoliation. One gram of CN was incorporated into a sample with 20 mL of concentrated sulfuric acid (H_2_SO_4_) and 20 mL of concentrated nitric acid (HNO_3_). Following this, the suspension was then magnetically stirred and refluxed at 80 °C for 2 h, and the reaction mixture was poured into 1 L of deionized water. The product was washed several times with deionized water until a nearly neutral pH was reached. The resultant was dispersed in 20 mL of deionized water and ultrasonicated for 2 h until a homogeneous CNNP suspension was obtained [12].

### 2.3. Green Synthesis of Ag and NiO Nanoparticles

Silver nanoparticles and nickel oxide nanoparticles were prepared via a green synthesis process, with plant extract as a reducing and stabilizing agent. The plant material was washed multiple times using distilled water, dried, and used for extract preparation. In Ag NPs synthesis, silver nitrate solution was combined with the plant extract and stirred continuously until an obvious color change suggested the formation of nanoparticles. The resulting suspension was centrifuged, thoroughly washed with distilled water several times, dried, and calcined to derive Ag nanoparticles. NiO nanoparticles were prepared by mixing nickel nitrate hexahydrate with the plant extract under heating until a green gel was formed. Then, the obtained gel was dried and calcined in a muffle furnace to form NiO nanoparticles. The prepared nanoparticles were stored in clean sample containers for further characterization and incorporation into PVC films [13].

### 2.4. Preparation of the CNNP/GO Hybrid Nanocomposite

For hybrid nanocomposite preparation, CNNP stock suspension containing a concentration of approximately 10 mg/mL was used. Here, to make a homogeneous suspension, 10 mg of graphene oxide (GO) was first dispersed in 50 mL of deionized water, followed by 1 h of ultrasonication. Then, a predetermined amount of the CNNP suspension was added gradually to the GO suspension, followed by ultrasonication for 1 h. The mixture was subsequently magnetically stirred for 12 h to allow for homogeneous distribution and interfacial interaction between the CNNPs and GO nanosheets [14]. The generated CNNP/GO suspension was then collected and used for PVC film preparation.

### 2.5. Preparation of PVC Nanocomposite Films

PVC (İzmir province of Turkey; K-value 67; degree of polymerization ≈800) was purified via repeated precipitation from THF using ethanol and dried under vacuum at room temperature for 24 h. The films were prepared via solvent casting. A total of 0.5 g of PVC was dissolved in 10 mL of THF and stirred for 1 h; CNNP/GO, Ag NPs, and NiO NPs were separately dispersed in 10 mL of THF by ultrasonication and gradually added to the PVC solution. The CNNP/GO loading was fixed at 0.5 wt%, while Ag NPs and NiO NPs were incorporated at two concentrations, 0.3 and 0.5 wt%. The mixtures were stirred for 2–3 h, poured into glass molds, and dried at room temperature for 24 h, followed by drying under reduced pressure. Blank PVC films were prepared without nanomaterials. The final film thickness was approximately 40 μm [15].

Comparable nanoparticle loadings were employed at 0.3 and 0.5 wt% concentrations, and these were not to be considered as optimum absolute levels. Instead, they were applied to explore the concentration-dependent photostabilization behavior of Ag NPs and NiO NPs at comparatively low nanofiller concentrations in the PVC matrix.

The nanoparticle concentrations were modest relative, not optimum, loadings, and the CNNP/GO films were set at 0.5 wt%, whereas the Ag and NiO nanomaterials were utilized at 0.3 and 0.5 wt% to study how nanoparticle type and content affect PVC photostability. The present study did not evaluate PVC gelation or fusing, since the films were solution-cast from THF and not melt-processed.

### 2.6. Accelerated Testing Technique

Subsequently, PVC films were irradiated with an accelerated QUV weathering tester equipped with UV-B 313 lamps (Philips, Germany). The radiation emitted by the lamps was between 290–360 nm, with maximum intensity observed at 313 nm. The film samples were placed parallel with one another at a distance of 25 cm from the light source, with the radiation incident vertical on their surfaces. Sample positions were periodically rotated to ensure uniform UV exposure [15].

### 2.7. Examination of PVC Photodegradation via the Weight-Loss Method

The photostability of PVC was evaluated by measuring the weight loss of the polymer during irradiation in the presence of stabilizers [15]. Weight loss was calculated using Equation (1):

(1) $$\mathrm{Weight loss}\left(\%\right)=\frac{W_{0}-W_{t}}{W_{0}}\times 100$$

where W_0_ is the PVC film weight before irradiation, and W_t_ is its weight after irradiation.

### 2.8. Examination of PVC Photodegradation via FTIR

FTIR spectroscopy was employed to investigate the photodegradation of the polymer films by monitoring the formation of carbonyl (C=O) and alkene (C=C) functional groups. The functional-group index (I_s_)(I_s_)(I_s_) was calculated using Equation (2):

(2) $$I_{s}=\frac{A_{s}}{A_{r}}$$

where A_r_ represents the absorbance of the reference peak, while A_s_ represents the absorbance of the investigated functional-group peak.

## 3. Results and Discussion

### 3.1. Characterization of Synthesized Compounds by UV–Visible Spectroscopy

**Scheme 1:**
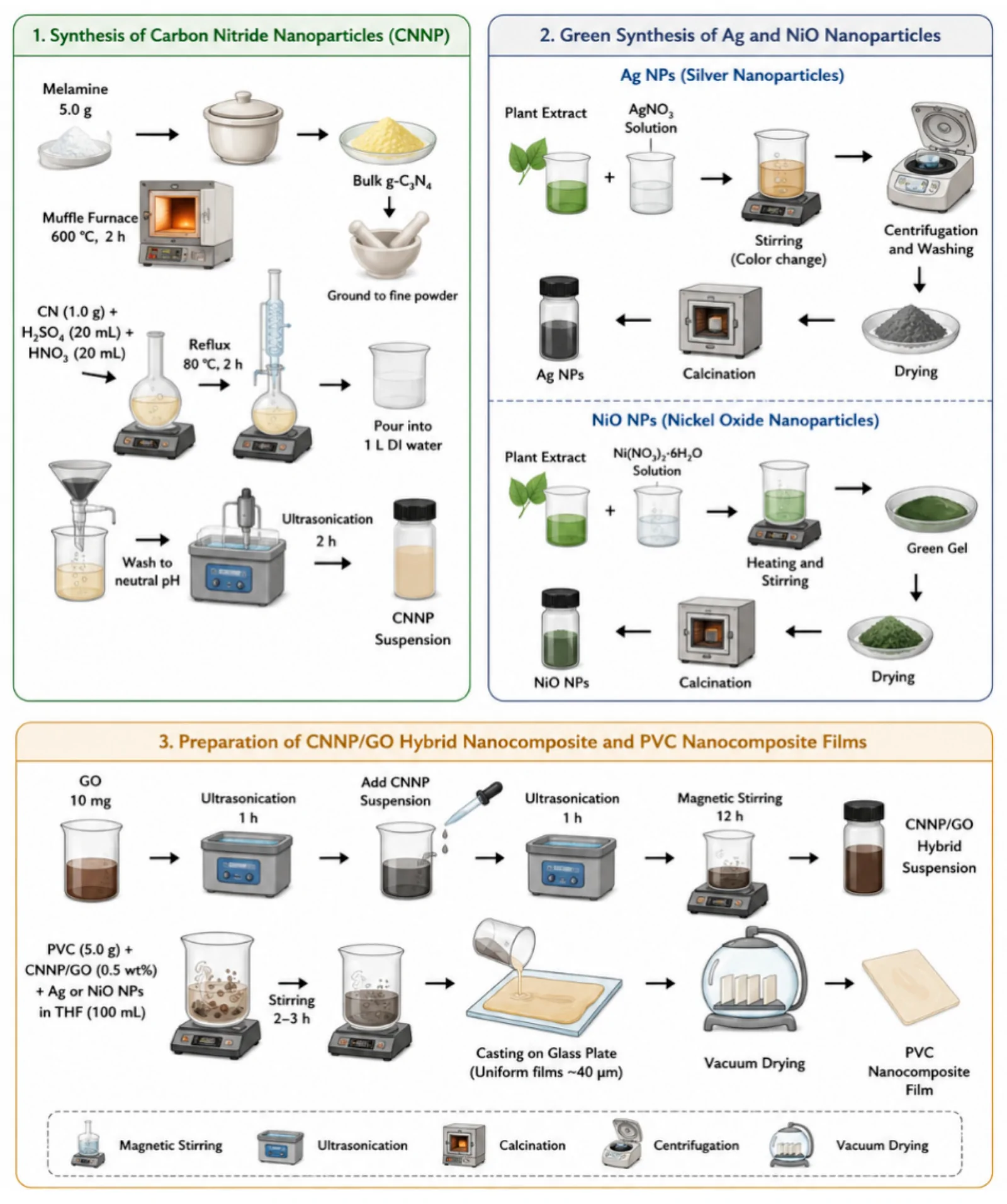
Methodology of preparation of PVC films.

**Figure 1:**
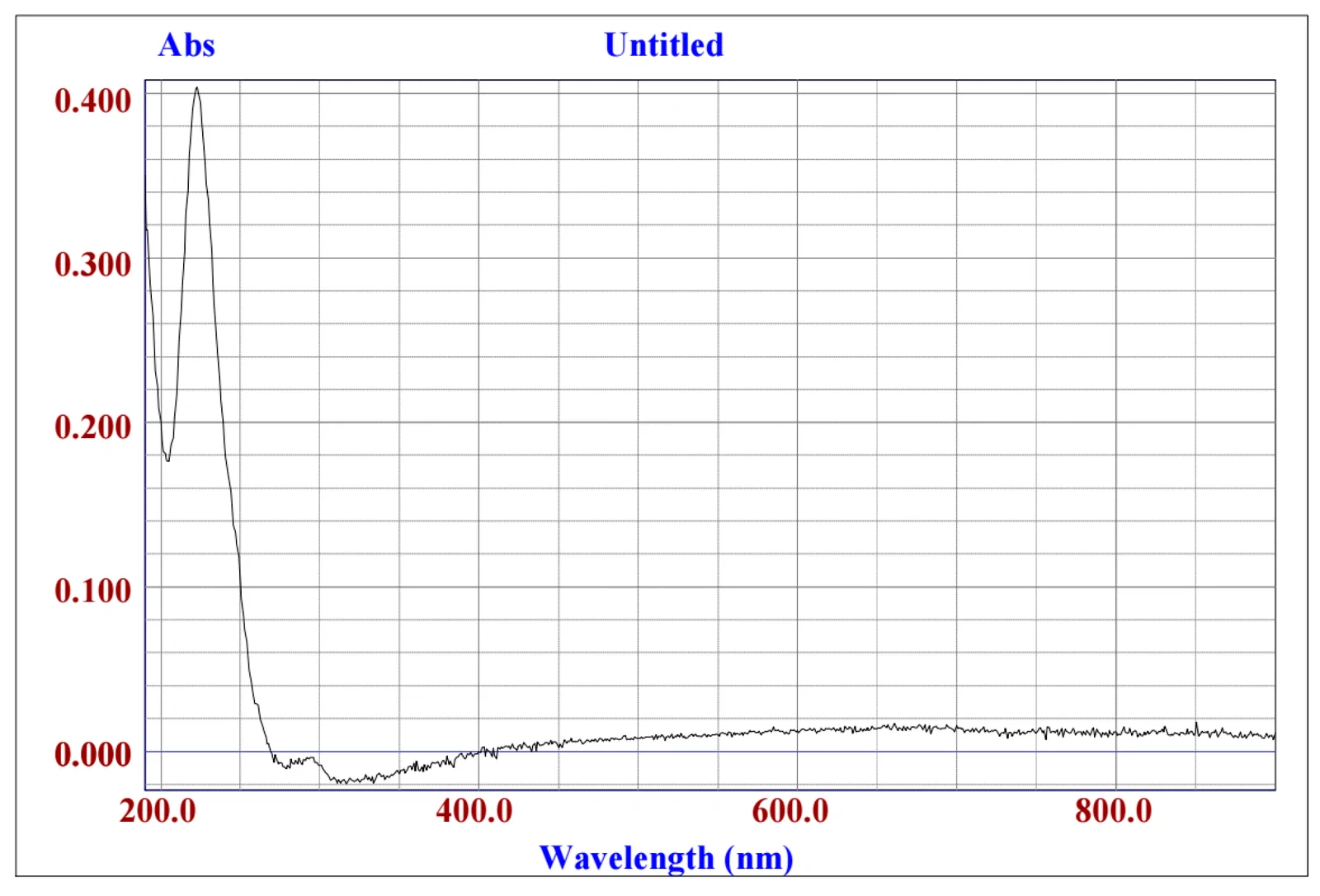
UV–Visible spectrum of synthesized CNNP.

**Figure 2:**
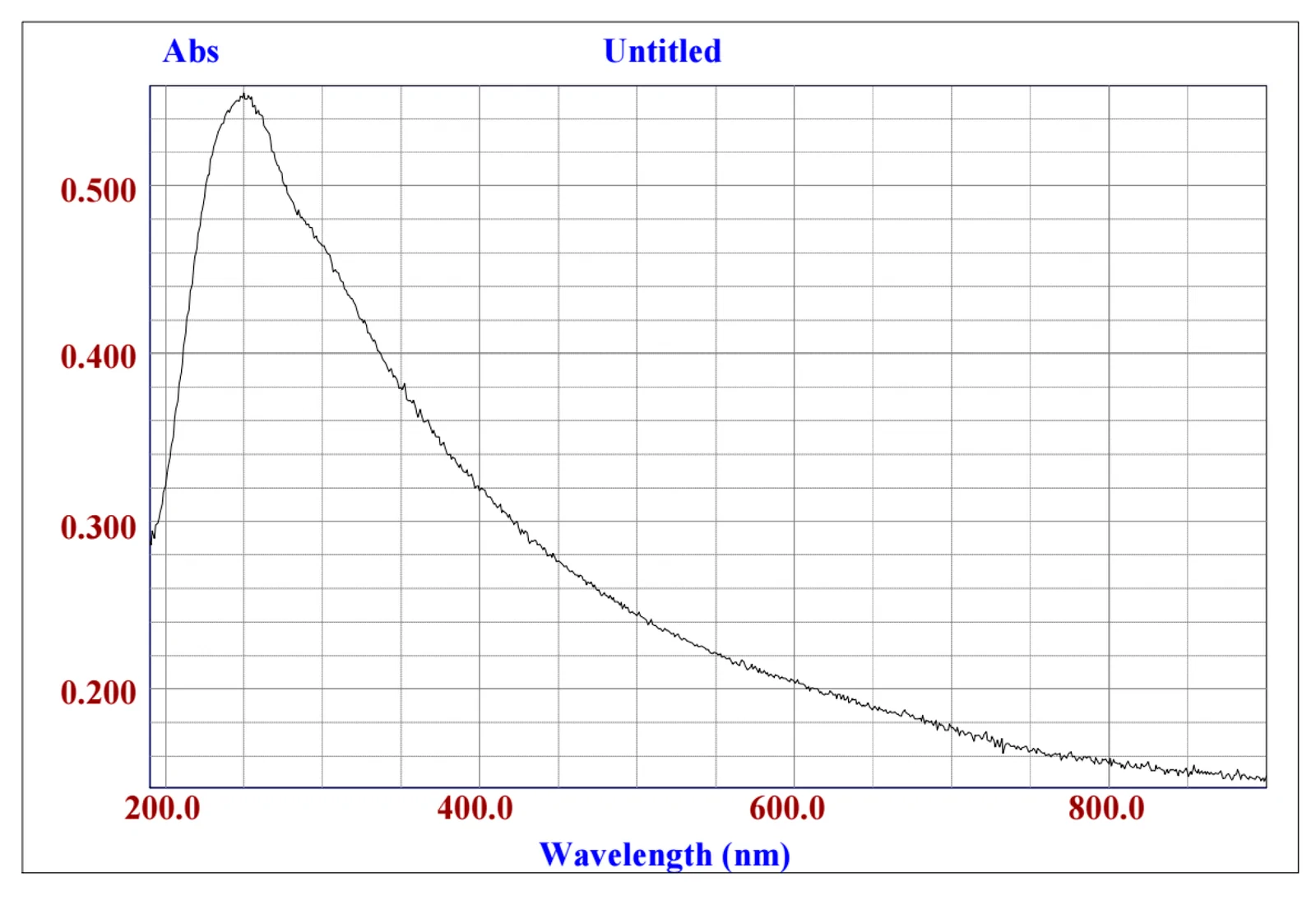
UV–Visible spectrum of synthesized GO.

The UV-Vis spectrum of CNNP revealed strong absorption below 300 nm, mainly attributed to π → π* transitions in the conjugated aromatic C–N framework of graphitic carbon nitride (Figure 1). The absorption tail extending to the visible spectrum can be attributed to electron delocalization, structural defects, and surface states, supporting the semiconducting nature of the prepared CNNP. The UV-Vis spectrum of GO showed a characteristic absorption band at 230–250 nm corresponding to π → π* transitions of aromatic C=C bonds (Figure 2). Moreover, a broad absorption tail extending into the visible region was also found and was ascribed to n → π* transitions in the hydroxyl, epoxy, carbonyl, and carboxyl oxygen-containing groups. These oxygenated functionalities enhanced the hydrophilic nature and aqueous dispersibility of GO, thus facilitating a homogeneous distribution of GO in nanocomposite preparation [16] (Scheme 1).

#### Analysis of Ag and NiO NPs Using UV-VIS Spectroscopy

**Figure 3:**
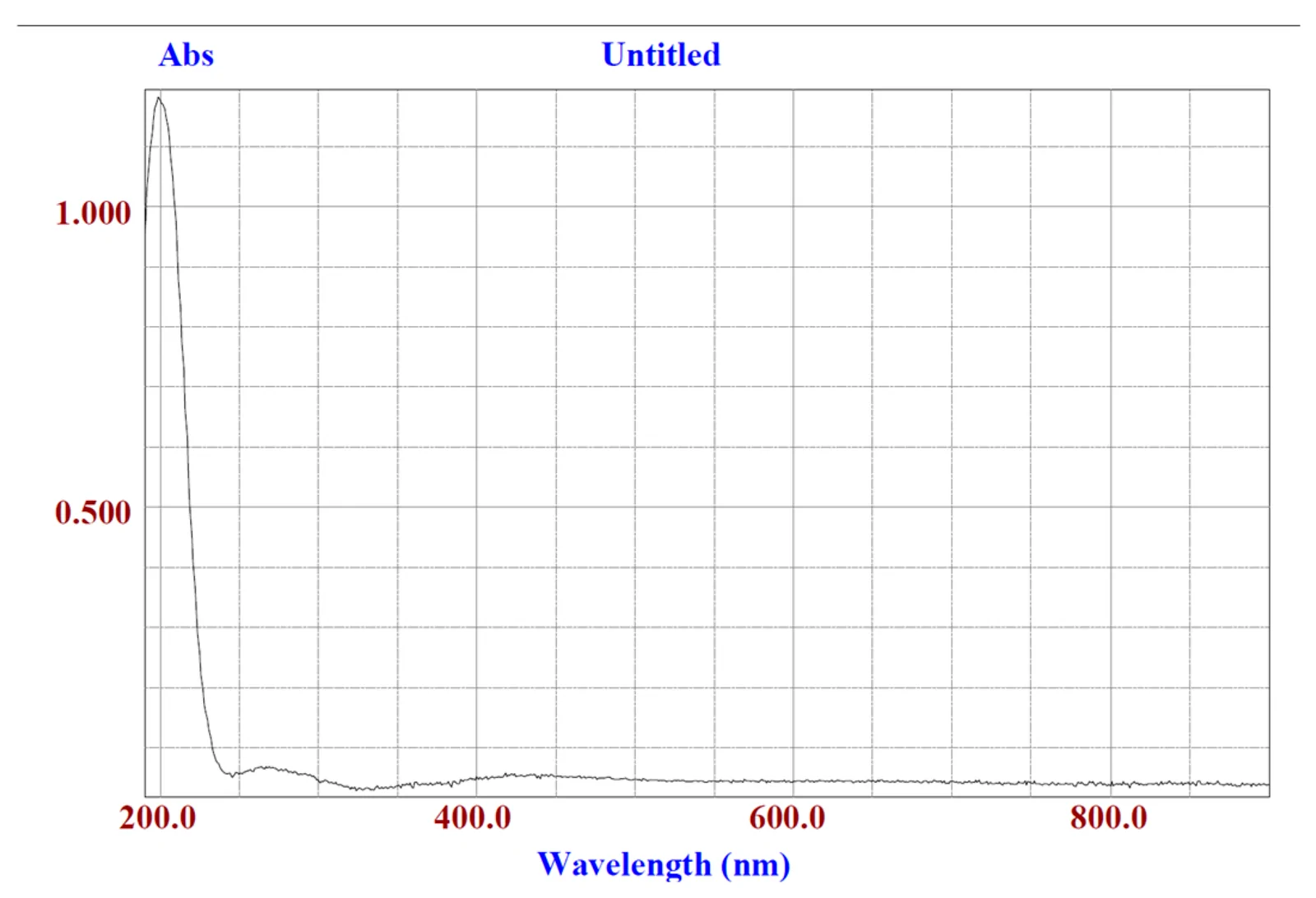
UV–Visible spectrum of Ag NPs.

**Figure 4:**
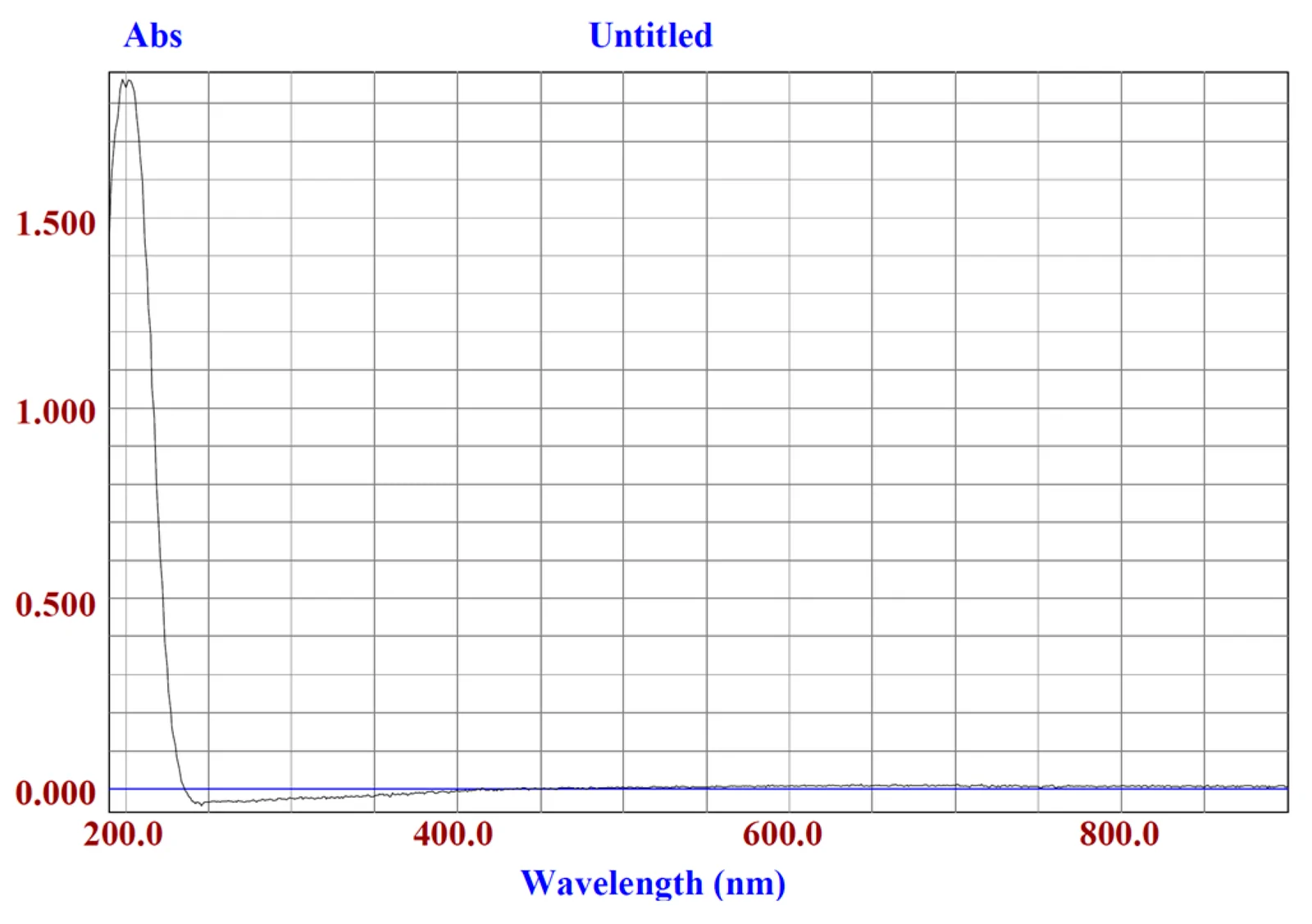
UV–Visible spectrum of NiO NPs.

The strong absorption band with Ag NPs was observed at approximately 210–230 nm, with relatively low absorption in the visible region (Figure 3). Since no clear SPR band appeared near 400–450 nm, XRD, TEM, and EDX confirmed Ag formation. High absorption of NiO NPs observed below 250 nm was ascribed to O^2−^ → Ni^2+^ charge-transfer transitions typical of NiO (Figure 4).

### 3.2. Characterization of Synthesized Compounds by FTIR

**Figure 5:**
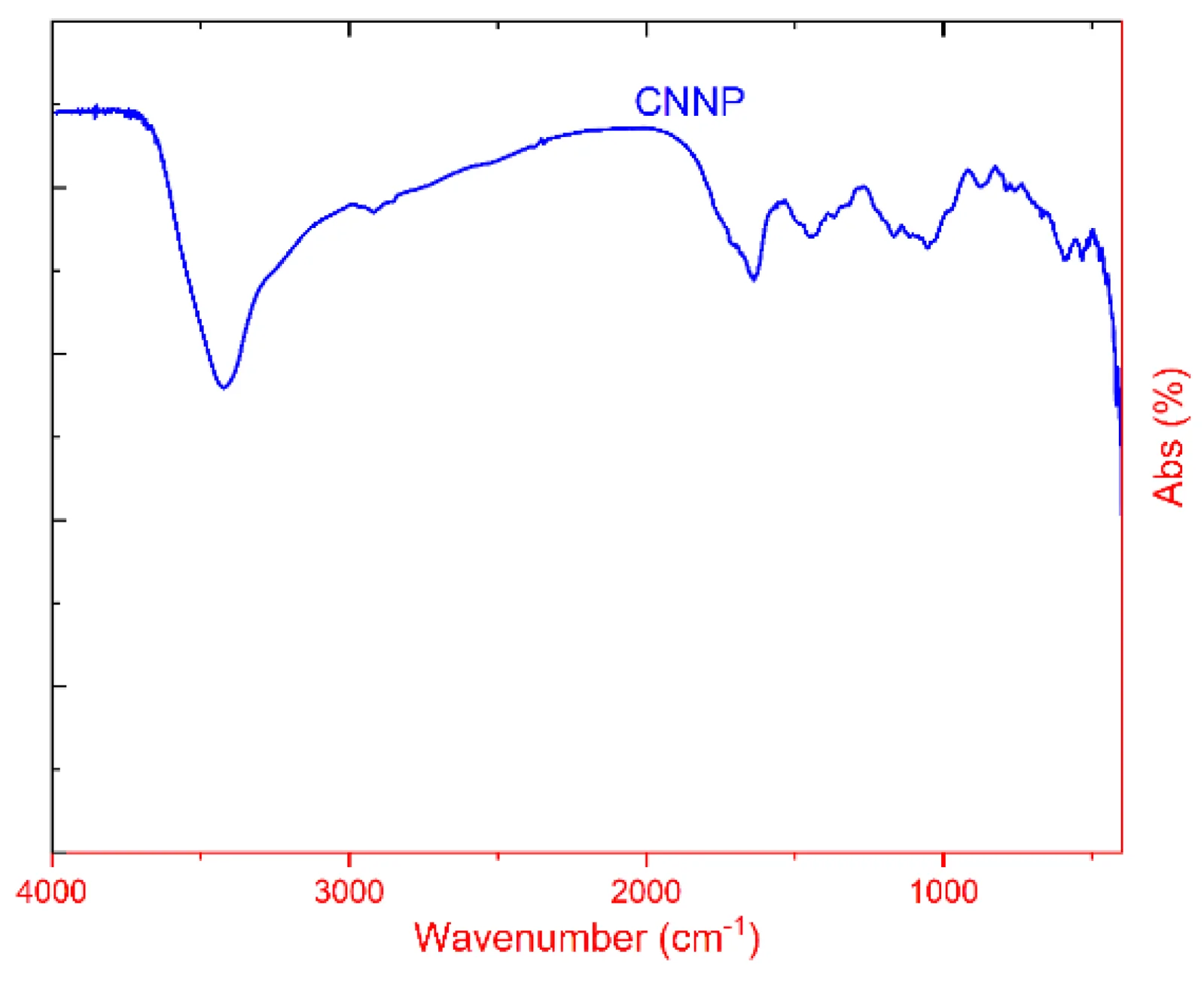
FTIR spectrum of CNNP.

**Figure 6:**
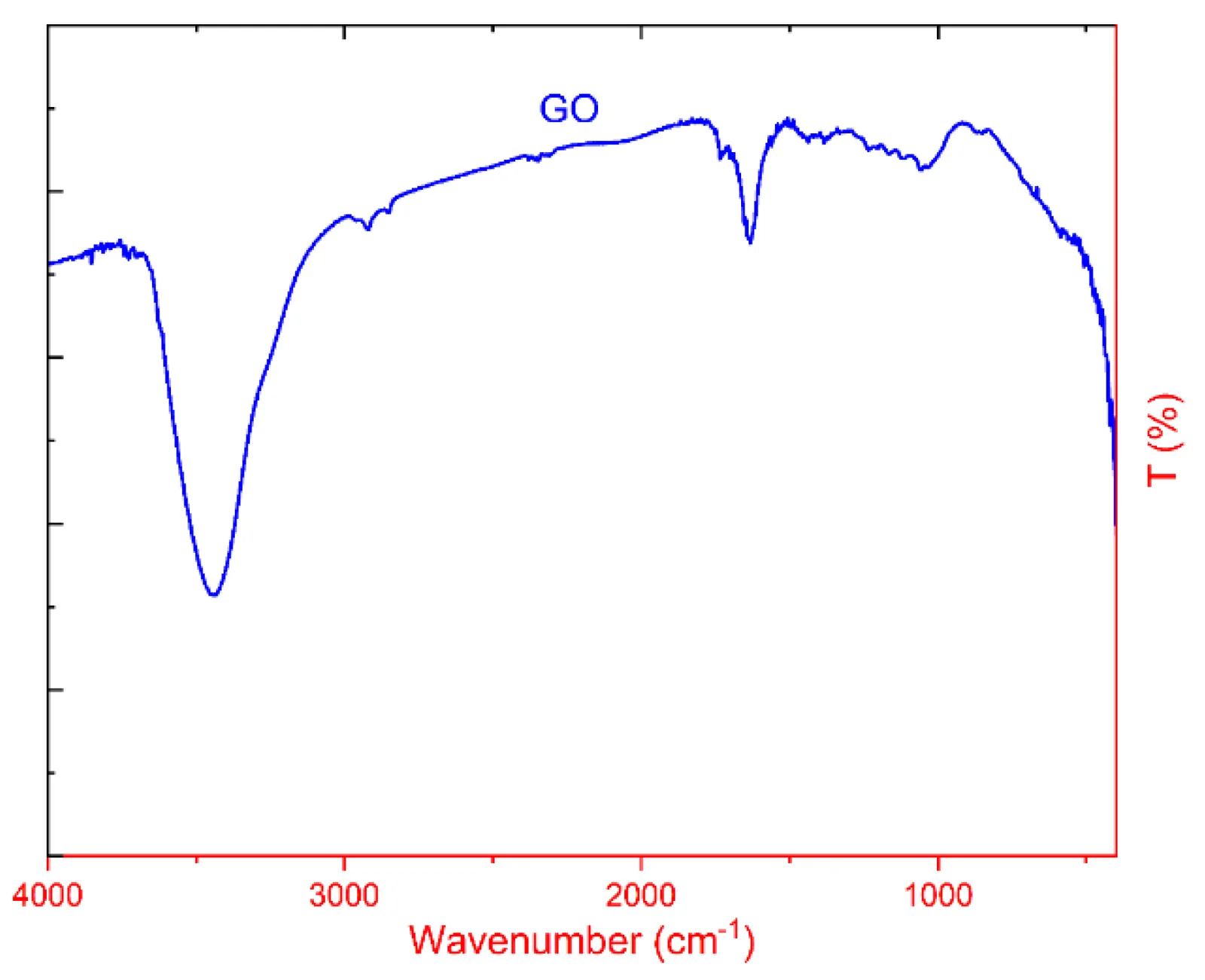
FTIR spectrum of GO.

The FTIR spectrum for CNNP presented a broad band near 3400 cm^−1^, attributed to the stretching of the N–H/O–H and adsorbed water. Vibrations between 1200 and 1700 cm^−1^ with the triazine/heptazine framework of C–N and C=N vibrations were recognized; the peak near 800 cm^−1^ was attributed to ring-breathing vibrations and confirmed the graphitic carbon nitride structure (Figure 5). The GO spectrum presented a broad O–H band at 3200–3500 cm^−1^, a C=O band near 1700 cm^−1^, and an aromatic C=C band around 1600 cm^−1^ [17]. The bands between 1000 and 1400 cm^−1^ were attributed to C–O-containing groups, indicating successful oxidation of graphite (Figure 6).

**Figure 7:**
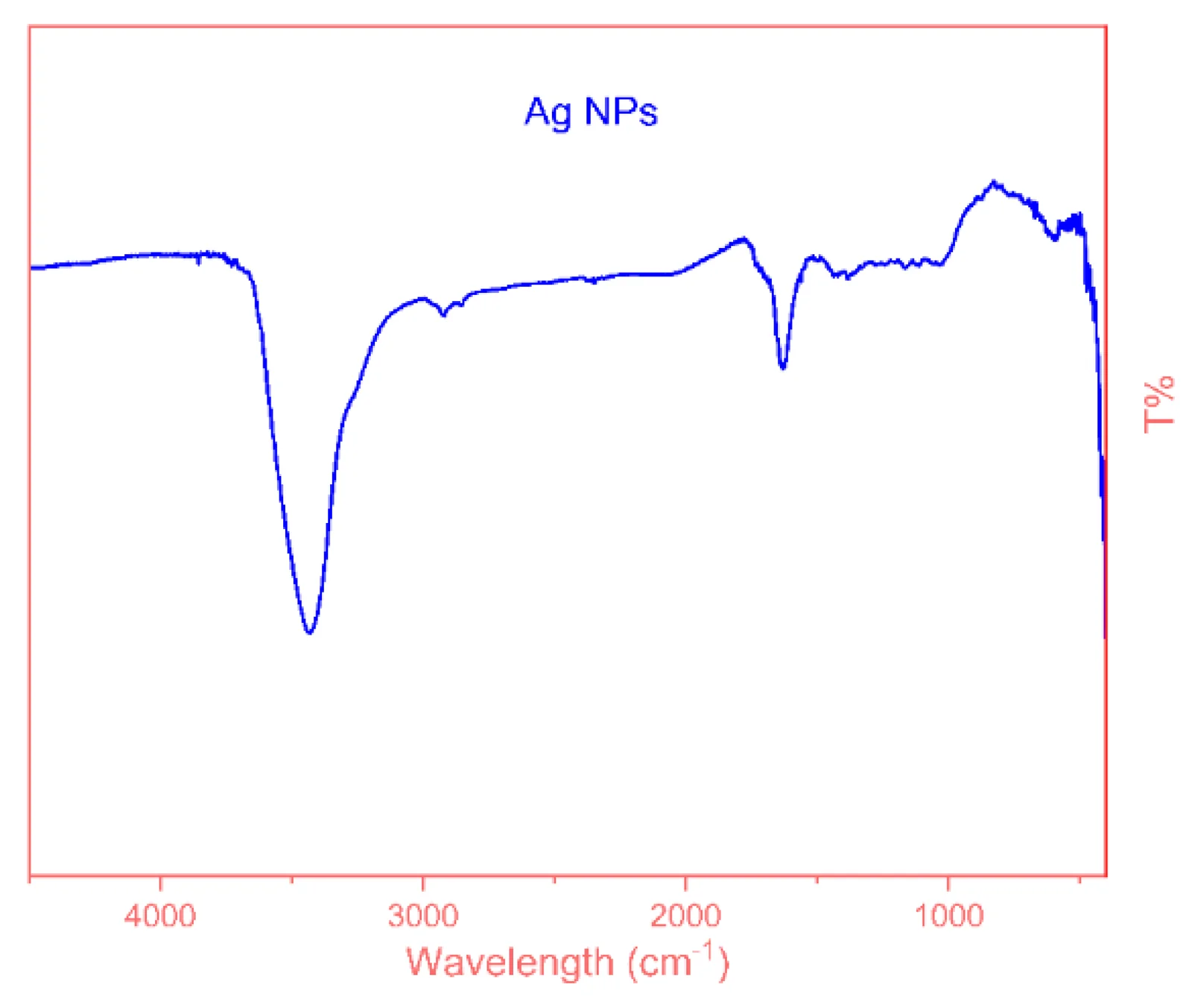
FTIR spectrum of Ag NPs.

The FTIR spectrum of Ag NPs showed a broad O-H band near 3429 cm^−1^ and a band around 1628 cm^−1^, attributed to carbonyl/amide groups from plant-derived compounds involved in nanoparticle stabilization. A low-frequency band near 419 cm^−1^ was assigned to metal-ligand vibrations (Figure 7).

**Figure 8:**
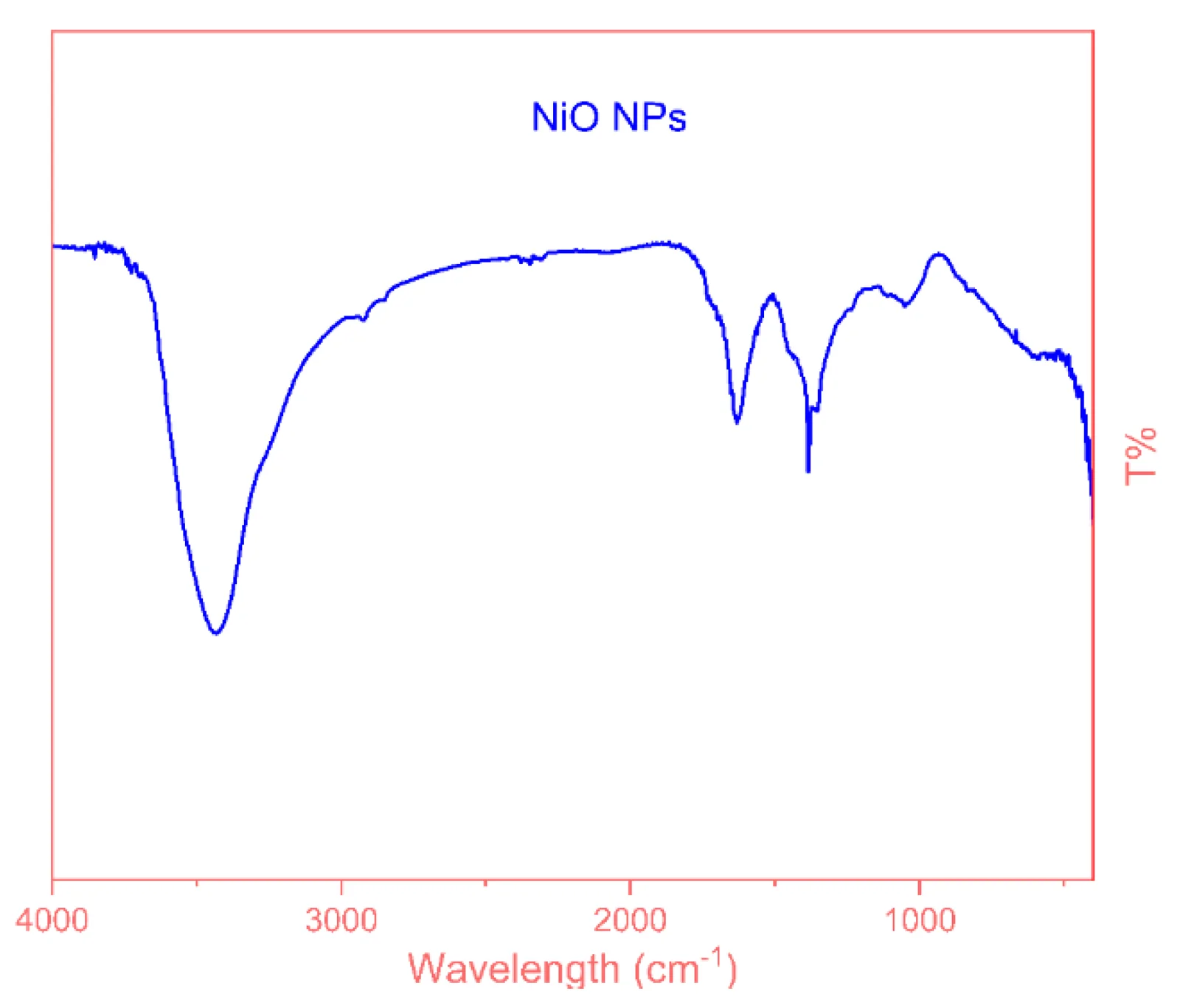
FTIR spectrum of NiO.

NiO NPs exhibited bands near 3431 and 1632 cm^−1^ due to surface hydroxyl groups and adsorbed water. The band around 1384 cm^−1^ was attributed to residual nitrate species [18], while the low-wavenumber region was associated with Ni-O vibrations, supporting NiO formation (Figure 8).

### 3.3. Characterization of Synthesized Compounds by FESEM and EDX

**Figure 9:**
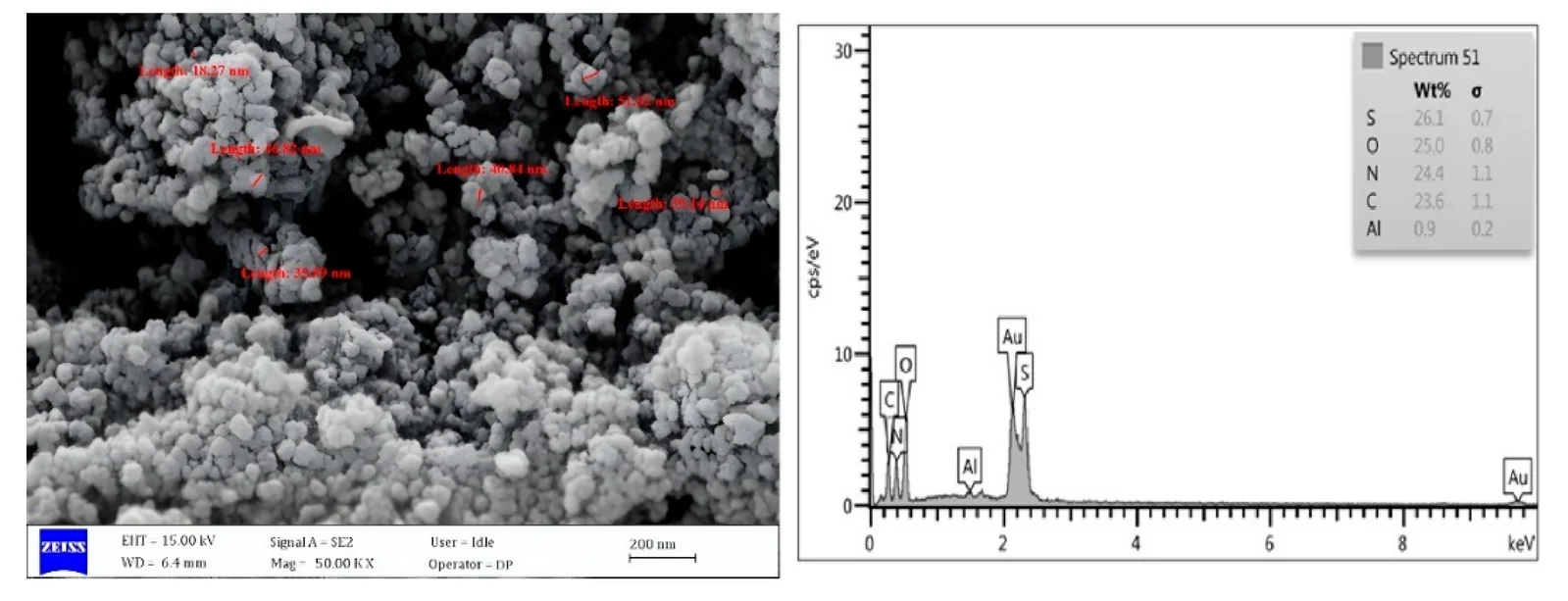
FESEM and EDX images of CNNP.

FESEM images showed that the nanoparticles were irregular and semi-spherical, with remarkable agglomeration associated with its high surface energy and interparticle interactions. Although clustered, they remained distributed fairly evenly over the large area of the images and were confirmed to form CNNPs, as shown in Figure 9.

**Figure 10:**
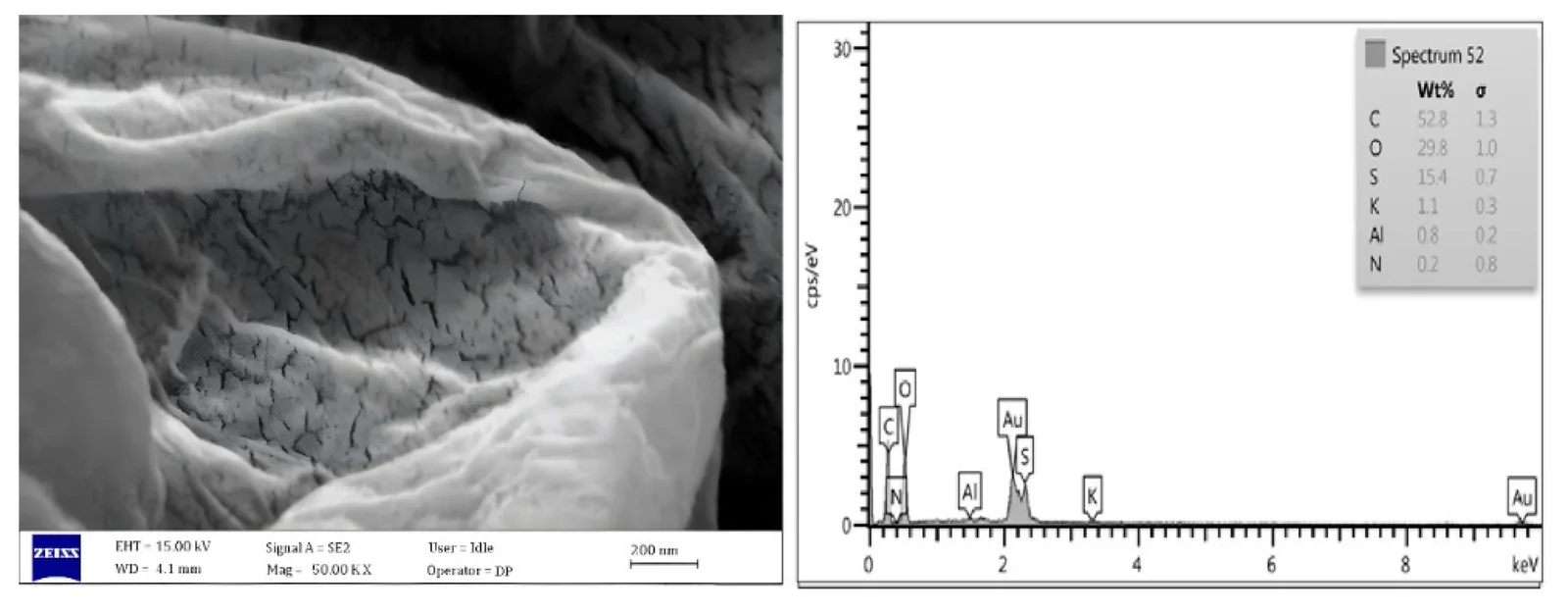
FESEM and EDX images of Go.

EDX analysis identified carbon and oxygen as the dominant elements. The high carbon content indicated the graphitic backbone, whereas oxygen suggested successful oxidation and the presence of oxygen-containing functional groups, as shown in Figure 10.

**Figure 11:**
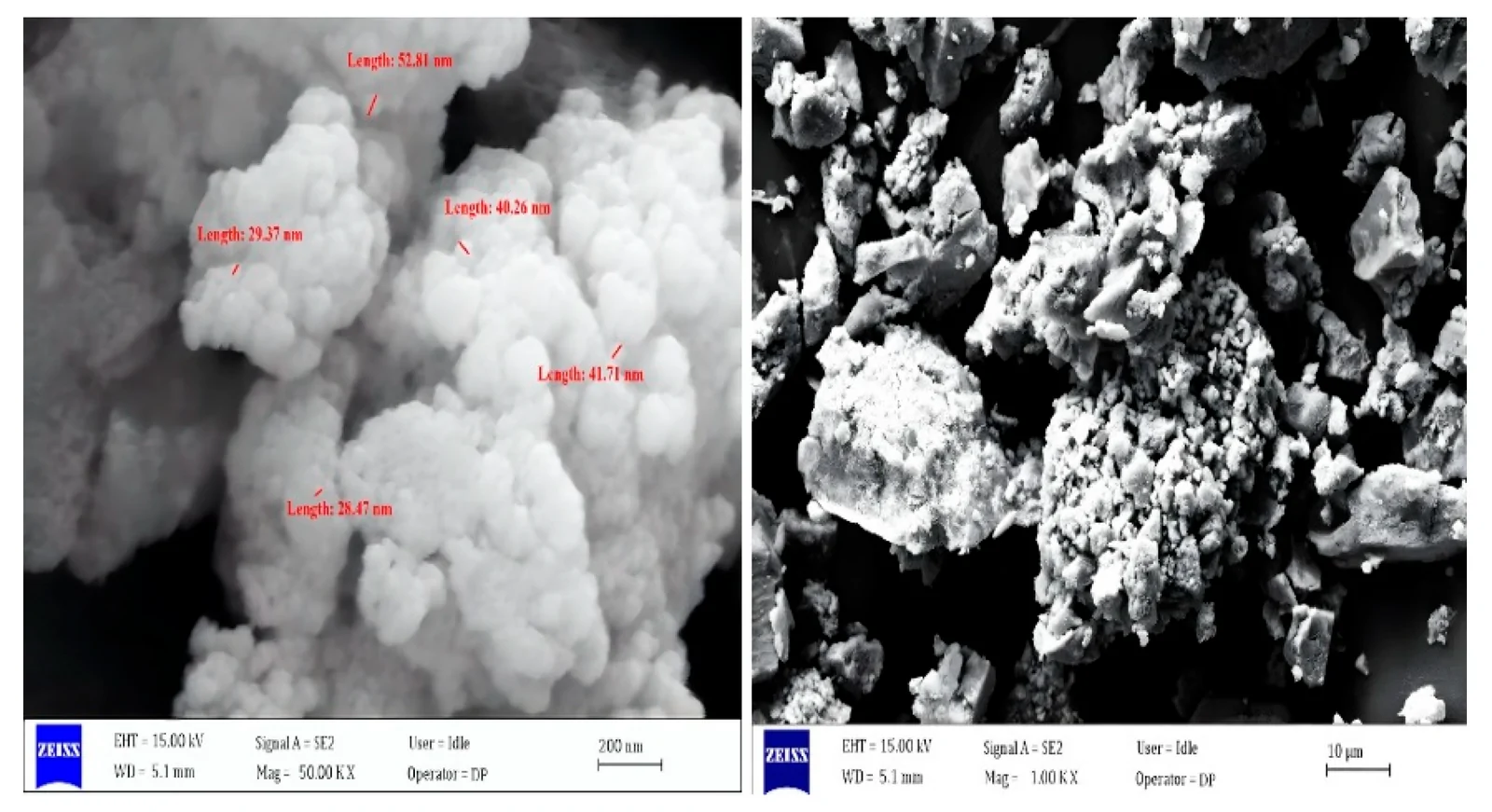
FESEM and EDX images of Ag NPs.

**Figure 12:**
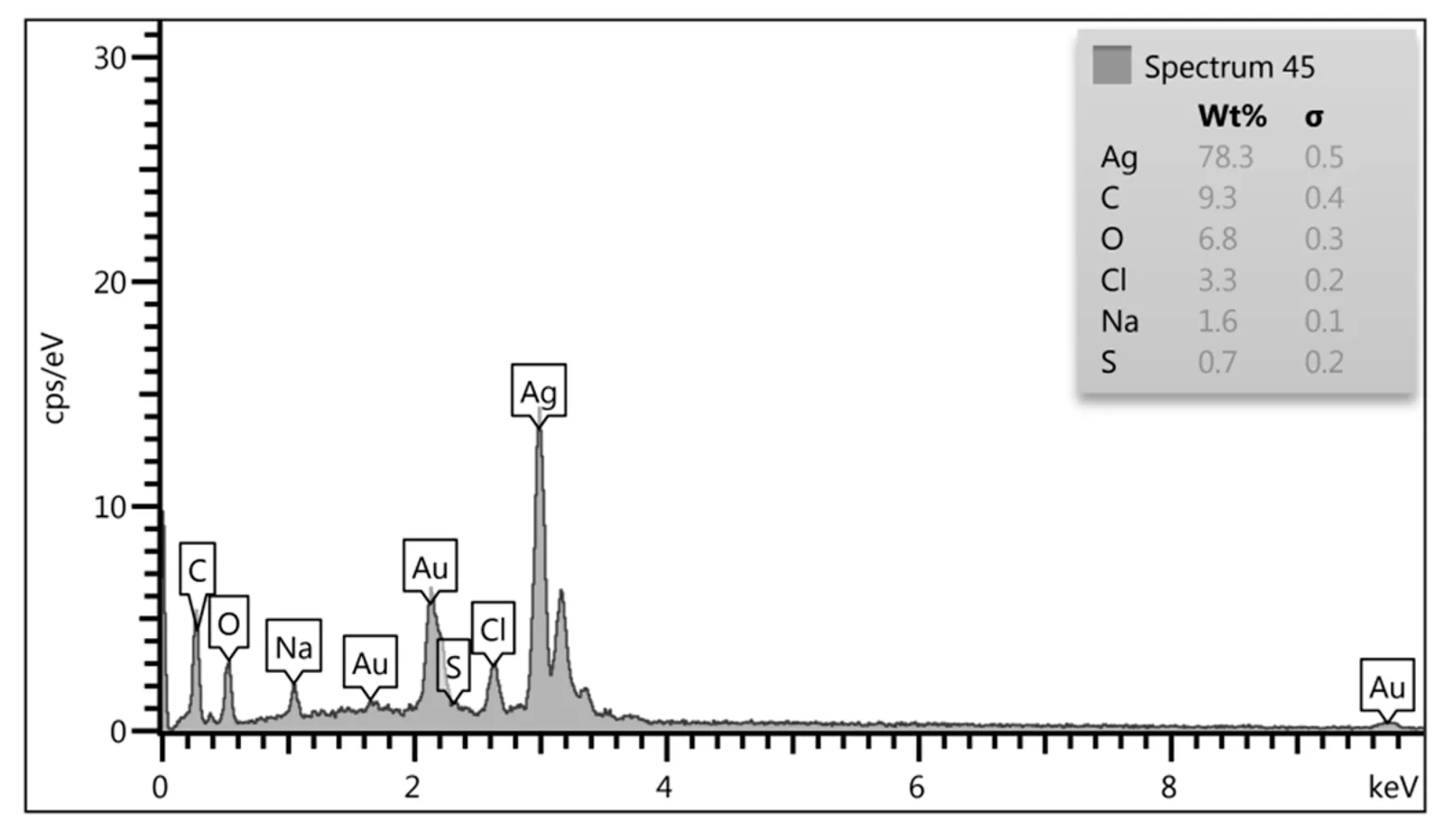
EDX spectrum of Ag NPs.

FESEM showed irregular to quasi-spherical Ag nanoparticles, with surface roughness and agglomeration. EDX exhibited a dominant Ag signal at ∼3 keV and 78.3 wt% silver value, and minor carbon or oxygen peaks were ascribed to residues of phytochemicals, as shown in Figure 11 and Figure 12.

**Figure 13:**
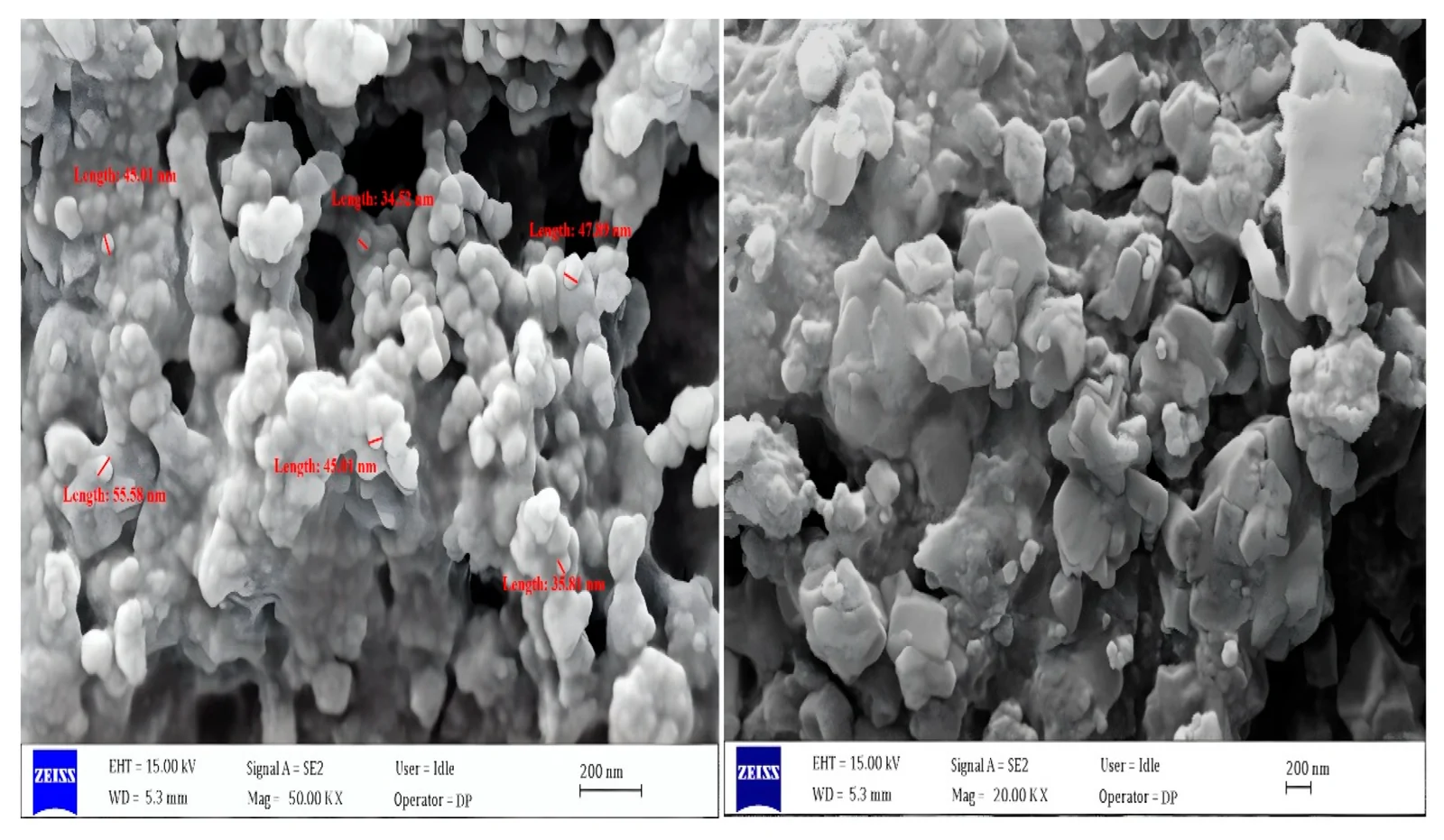
FESEM images of NiO NPs.

**Figure 14:**
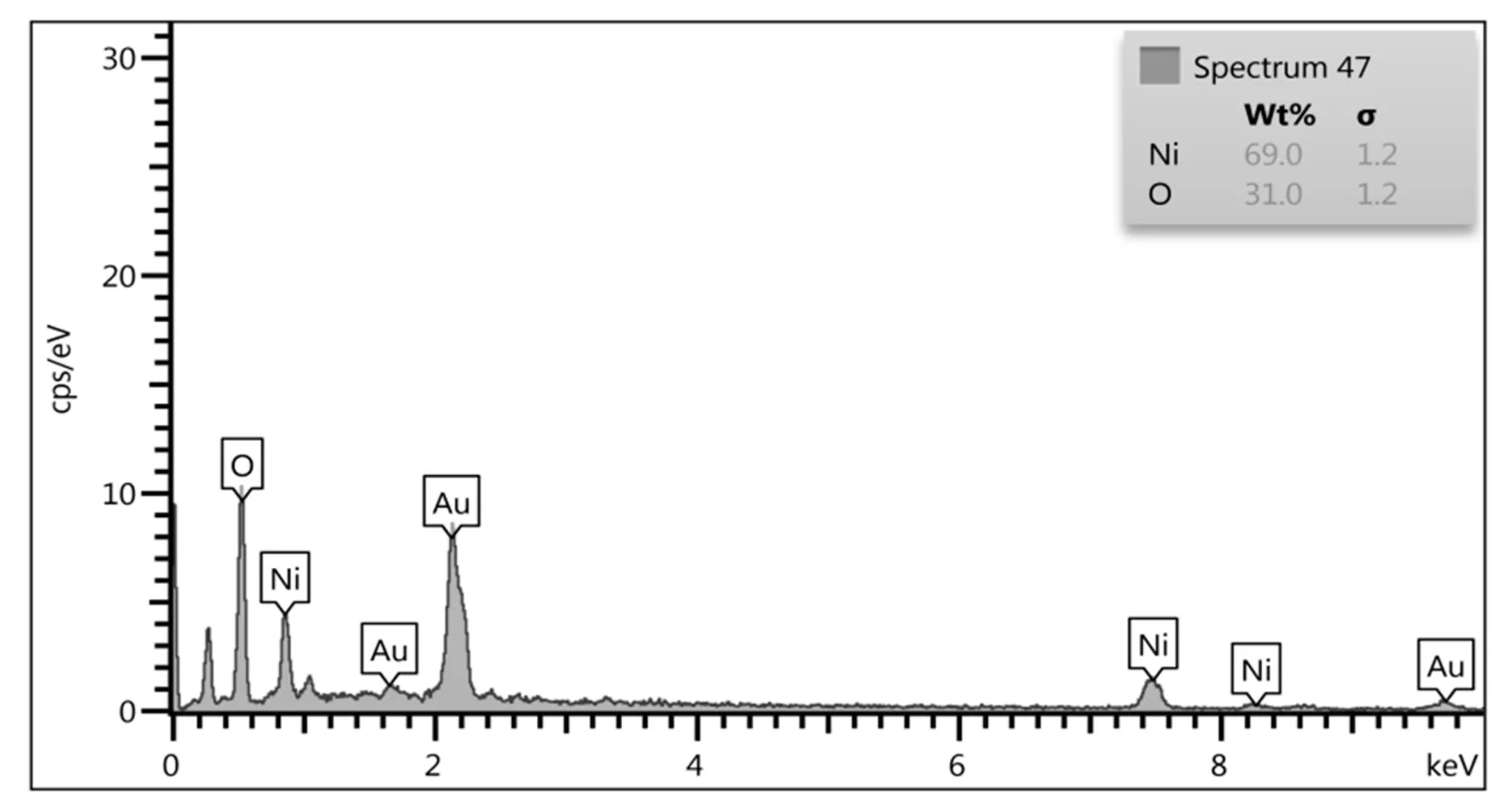
EDX spectrum of NiO NPs.

FESEM images showed agglomerated, heterogeneous, quasi-spherical to irregular particles with rough and porous surfaces. EDX confirmed NiO formation, with nickel and oxygen contents of 69.0 and 31.0 wt%, respectively, as shown in Figure 13 and Figure 14.

### 3.4. Characterization of Synthesized Compounds by TEM

**Figure 15:**
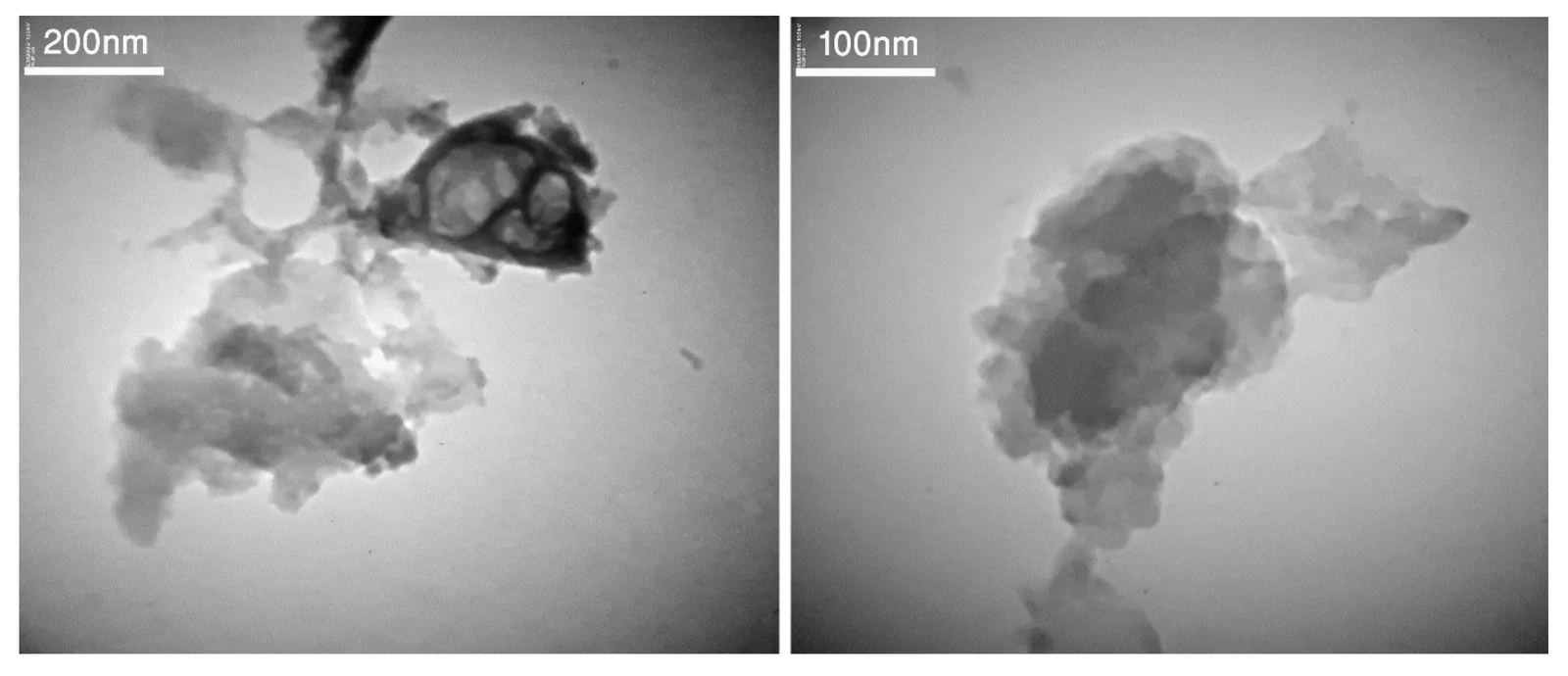
TEM analysis of CNNP.

TEM images revealed agglomerated nanoscale particles with irregular morphology. Partially transparent regions indicated thin carbon nitride layers, which verified successful CNNP formation, as shown in Figure 15.

**Figure 16:**
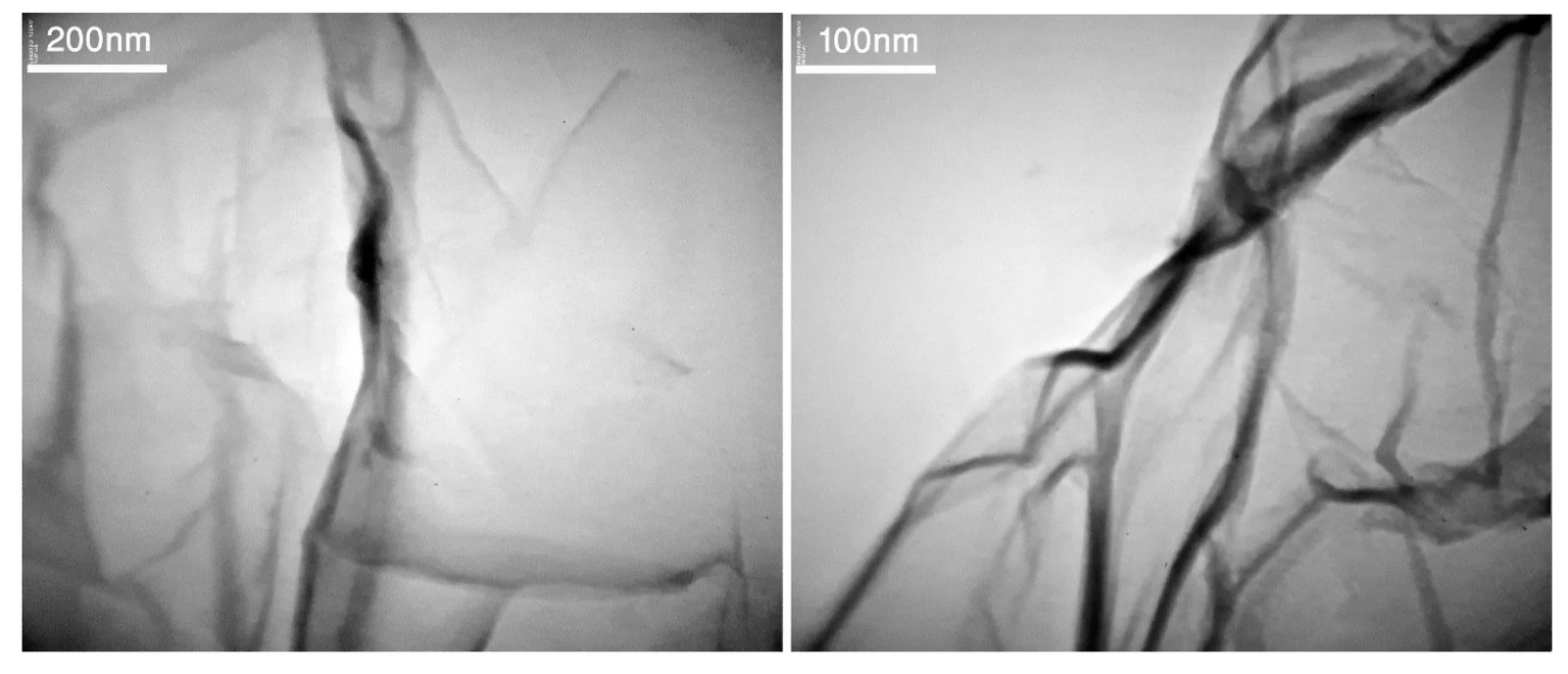
TEM analysis of GO.

Thin, transparent, wrinkled, and folded nanosheets visible on TEM indicated the oxidation and exfoliation of graphite to graphene oxide, as shown in Figure 16.

**Figure 17:**
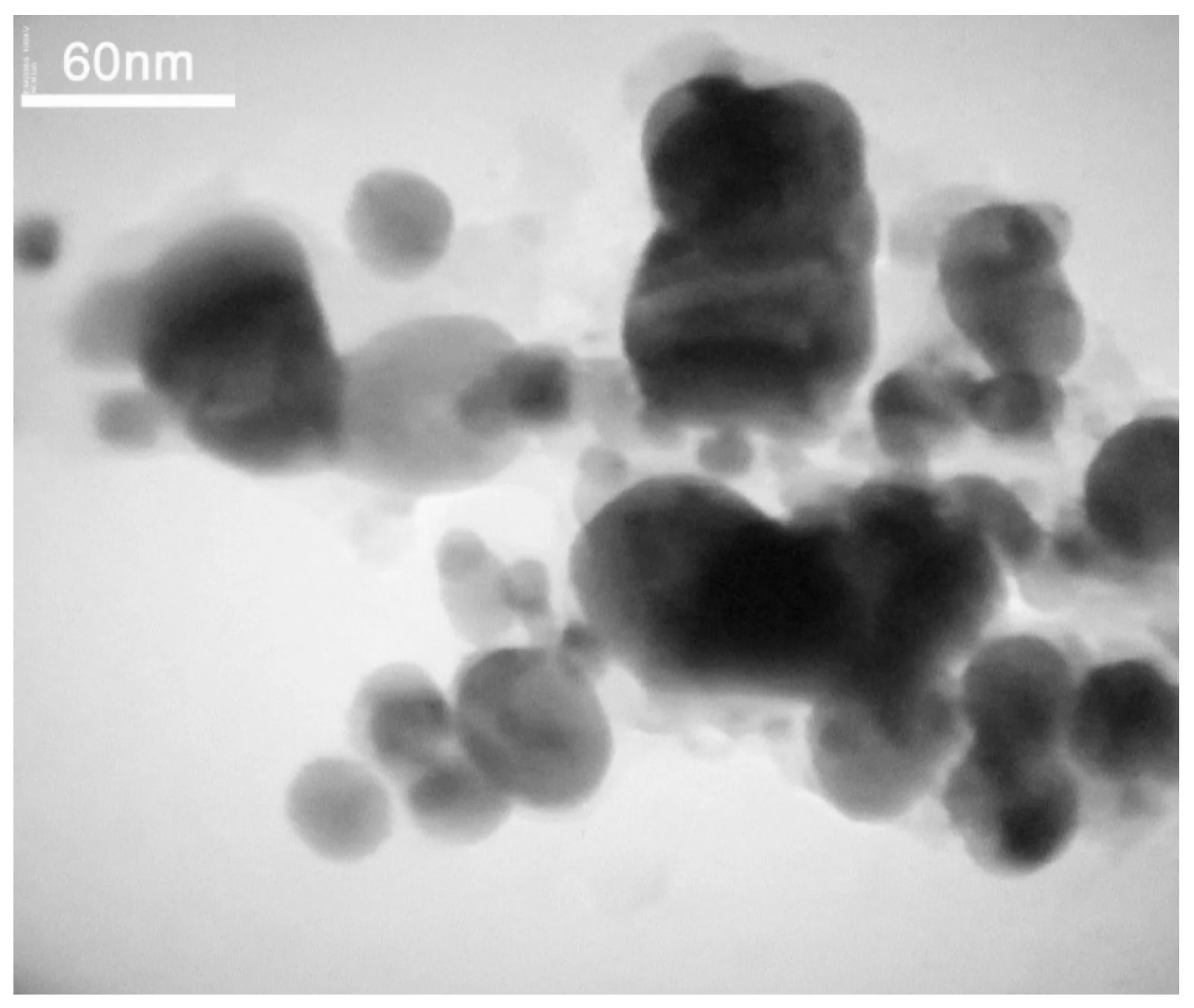
TEM analysis of Ag NPs.

TEM images of Ag NPs revealed spherical to quasi-spherical particles with local aggregation. The mean particle diameter was 36.35 ± 16.53 nm, and the median diameter was 36.20 nm in a quantitative study of 93 distinct primary particles, as shown in Figure 17.

**Figure 18:**
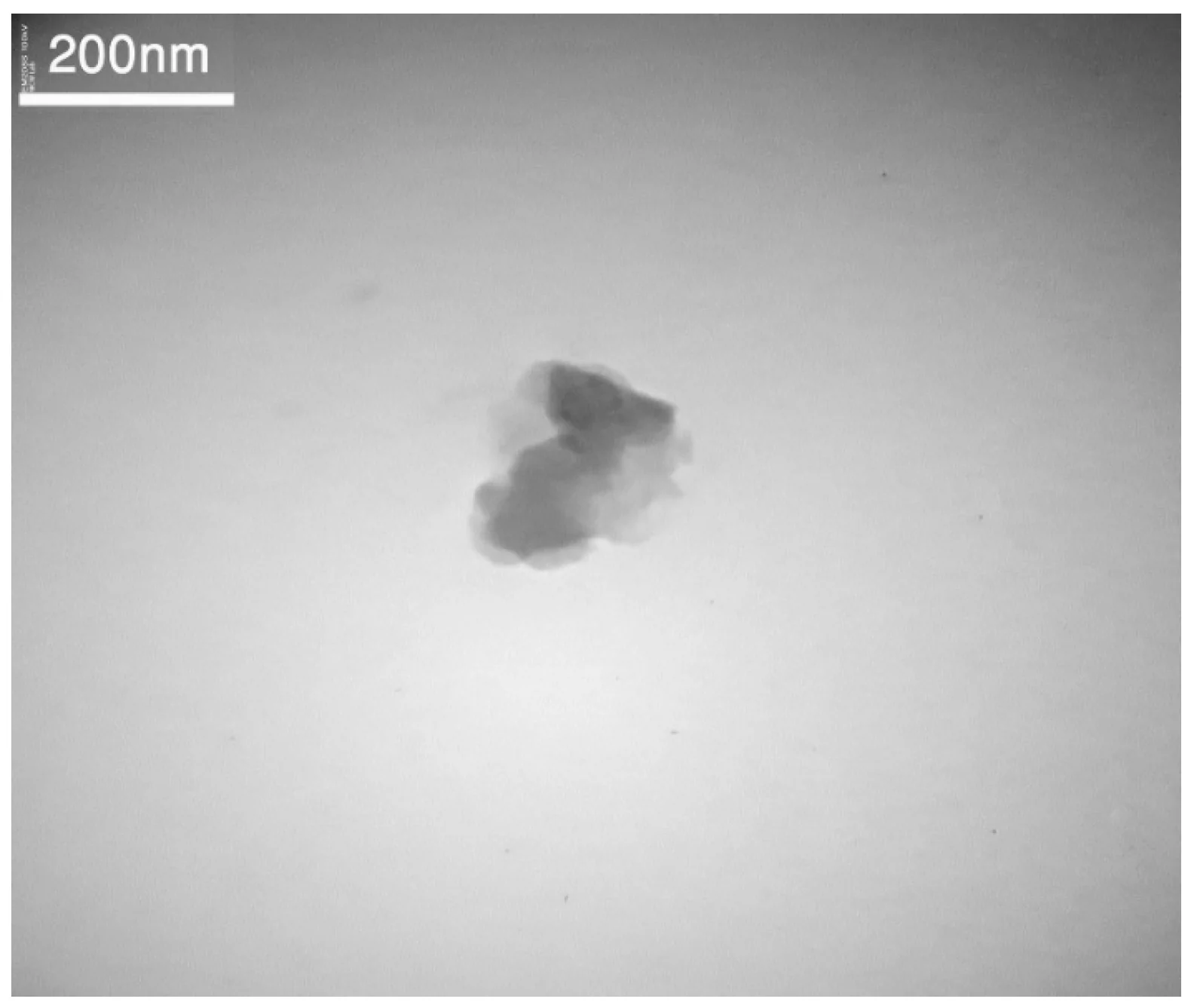
TEM analysis of NiO NPs.

TEM micrographs showed high-aggregation, irregular nanosized NiO particles, and the major particles had an average equivalent particle size of 29.9 ± 14.3 nm. NiO has a non-spherical shape; hence, the value is an equivalent particle dimension, not a diameter, as shown in Figure 18.

### 3.5. Monitoring the Photodegradation of PVC Films by FTIR Spectroscopy

**Figure 19:**
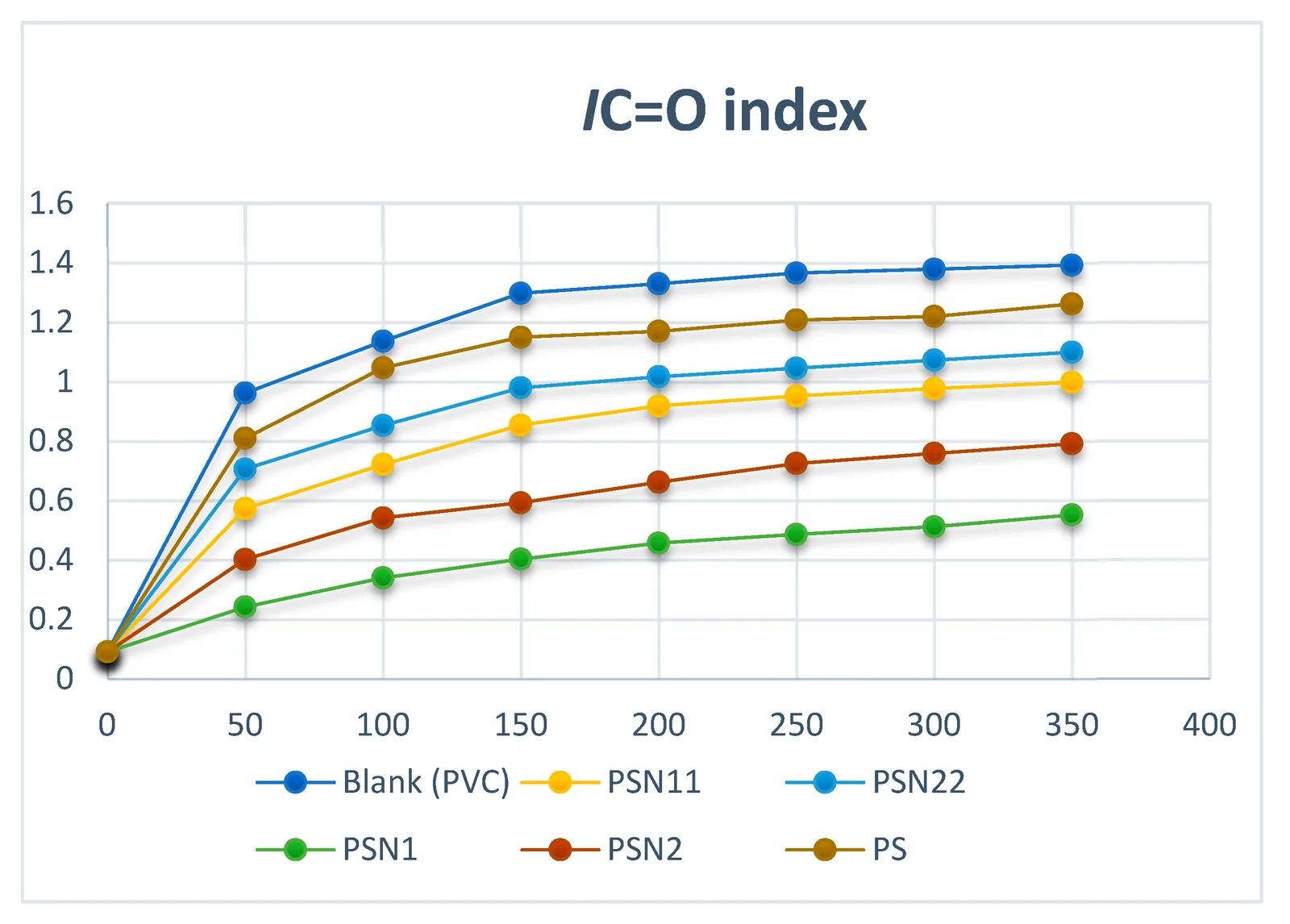
Effect of irradiation of PVC films on I_C=O_.

The carbonyl index showed an increase in all PVC films with irradiation time, which indicates progressive photooxidation. After 300 h, the blank PVC value was highest at 1.392 and lowest at 0.551 for PSN1, showing a reduction of approximately 60% compared to the blank, and PSN2 yielded the second lowest value at 0.791, followed by PSN11 at 0.998, PSN22 at 1.099, and PS at 1.261. Thus, the stabilization efficiency was PSN1 > PSN2 > PSN11 > PSN22 > PS > Blank. Accordingly, PSN1 was the best photostabilized film, as shown in Figure 19.

**Figure 20:**
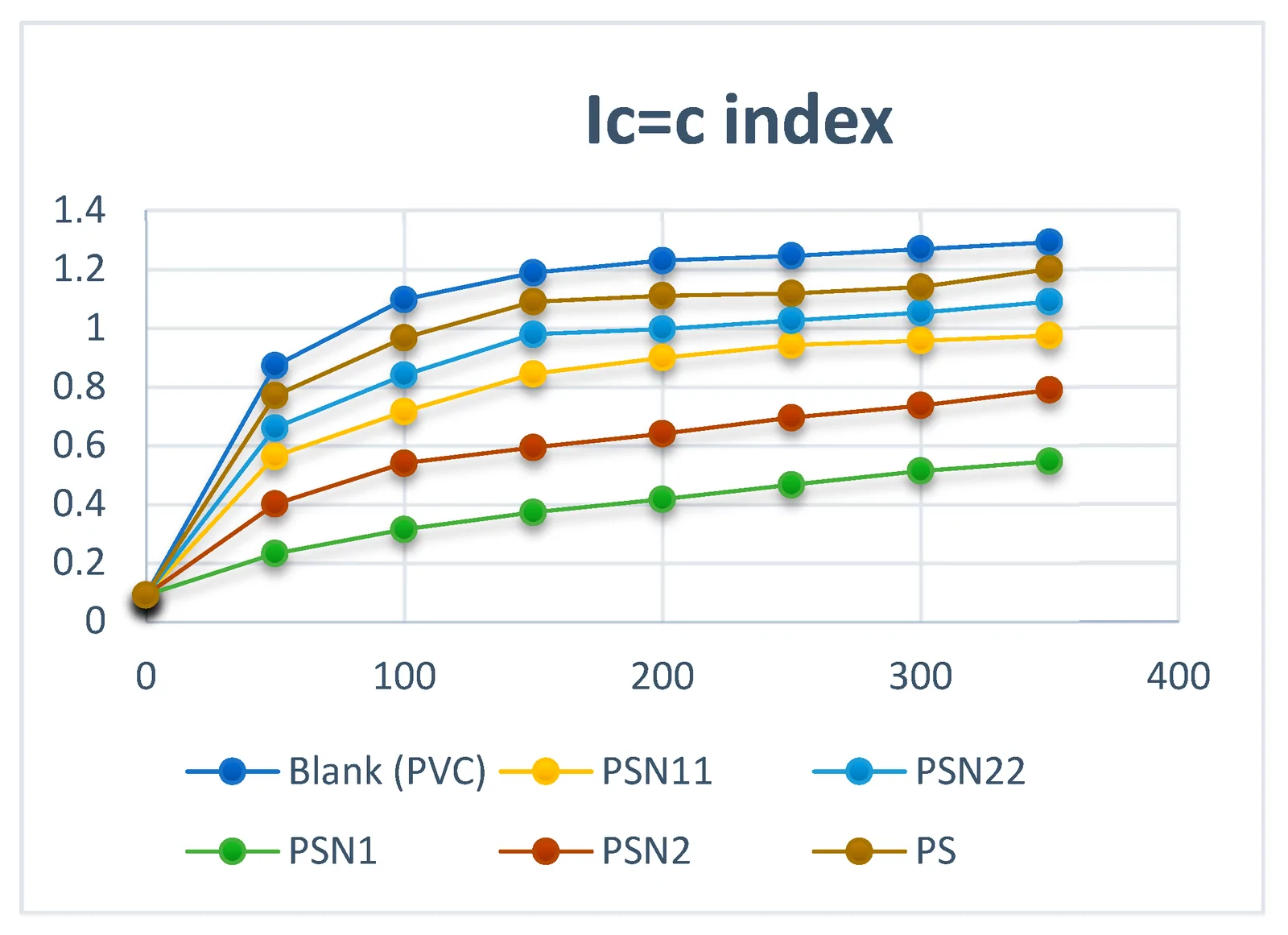
Effect of irradiation of PVC films on I_C=C_.

This also means that the polyene index increased with the number of hours of irradiation in PVC films, which reflects degradation in dehydrochlorination and the formation of conjugated C=C bonds. After 300 h, blank PVC yielded the highest value (1.292), and PSN1 returned the lowest value (0.545), which represents an approximately 58% decrease compared to levels for the blank film. At 350 h, the values were PSN1 (0.545) < PSN2 (0.789) < PSN11 (0.974) < PSN22 (1.089) < PS (1.201) < Blank (1.292). Thus, PSN1 was the most effective film in suppressing polyene production and PVC photodegradation, as shown in Figure 20.

**Figure 21:**
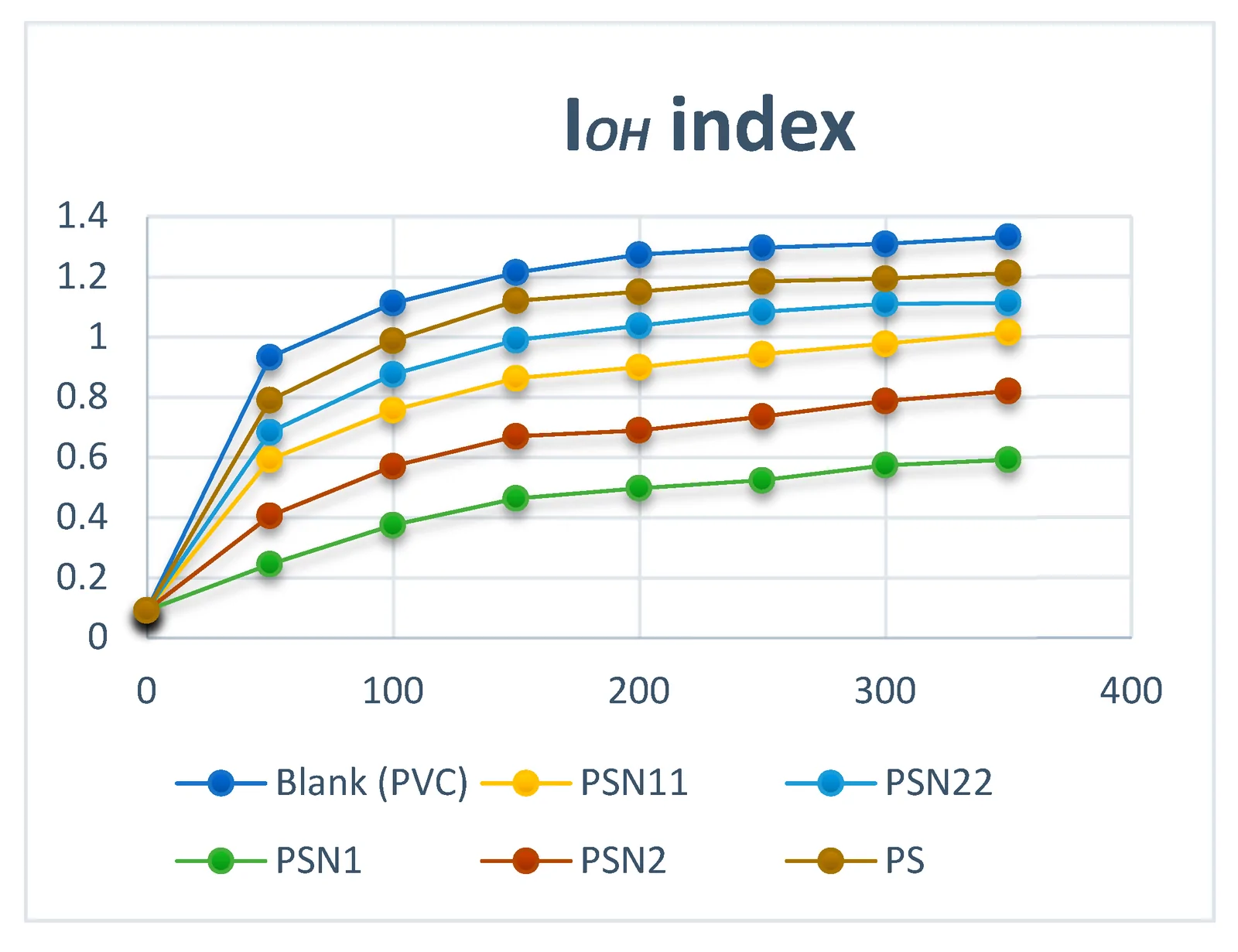
Effect of irradiation of PVC films on I_OH_.

The hydroxyl index enhanced with irradiation time for all PVC films, implying the evolution of photooxidation and hydroxyl-containing degradation products. After 350 h, blank PVC achieved the highest value (1.332) and PSN1 the lowest value (0.591), a nearly 56% improvement compared to the results for the blank. At 350 h, the values were PSN1 (0.591) < PSN2 (0.819) < PSN11 (1.014) < PSN22 (1.112) < PS (1.211) < Blank (1.332). Hence, PSN1 displayed the highest photostabilization efficiency at the site of UV irradiation through the effective suppression of hydroxyl-group formation, as shown in Figure 21.

### 3.6. Monitoring the Photodegradation of PVC Films via the Weight Loss Method

**Figure 22:**
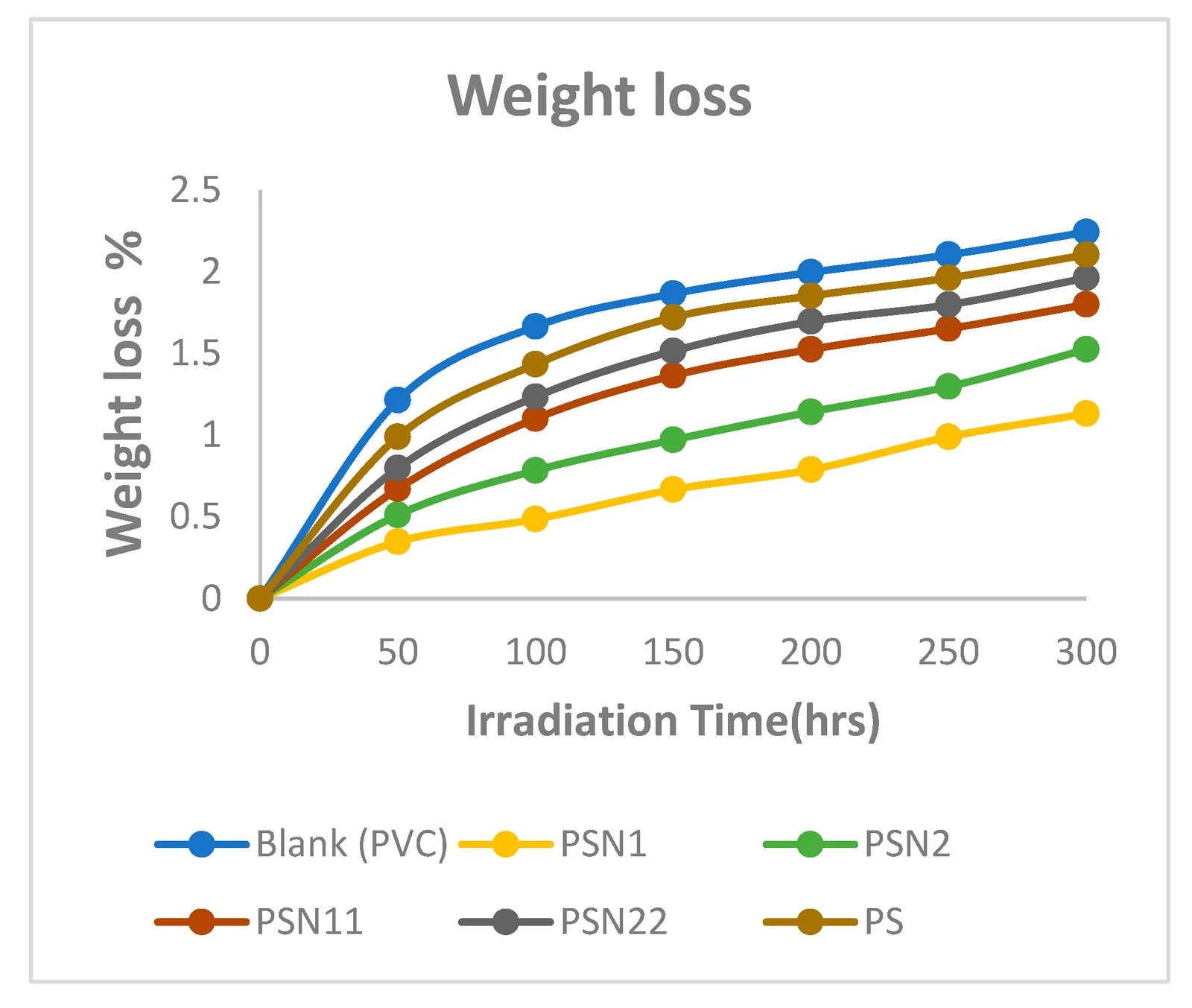
Weight loss of PVC films as a function of UV irradiation time.

The loss of weight had a positive relationship with irradiation time across all PVC films, indicating a progressive degradation and volatile loss. After 300 h, the blank PVC showed the highest weight loss (2.242%), followed by the lowest PSN1 (1.131%) (49.6% reduction compared with the blank). At 300 h, the values were PSN1 (1.131%) < PSN2 (1.525%) < PSN11 (1.799%) < PSN22 (1.961%) < PS (2.104%) < Blank PVC (2.242%). However, PSN1 exhibited the best protection against UV-induced mass loss, being the most efficient photostabilized film, as shown in Figure 22.

The dehydrochlorination of PVC, which releases HCl, as well as the photooxidative chain scission’s generation and the loss of other low-molecular-weight volatile compounds, reduce film mass under UV irradiation. Thus, higher weight-loss values indicate more polymer breakdown.

### 3.7. Polymer Surface Analysis

**Figure 23:**
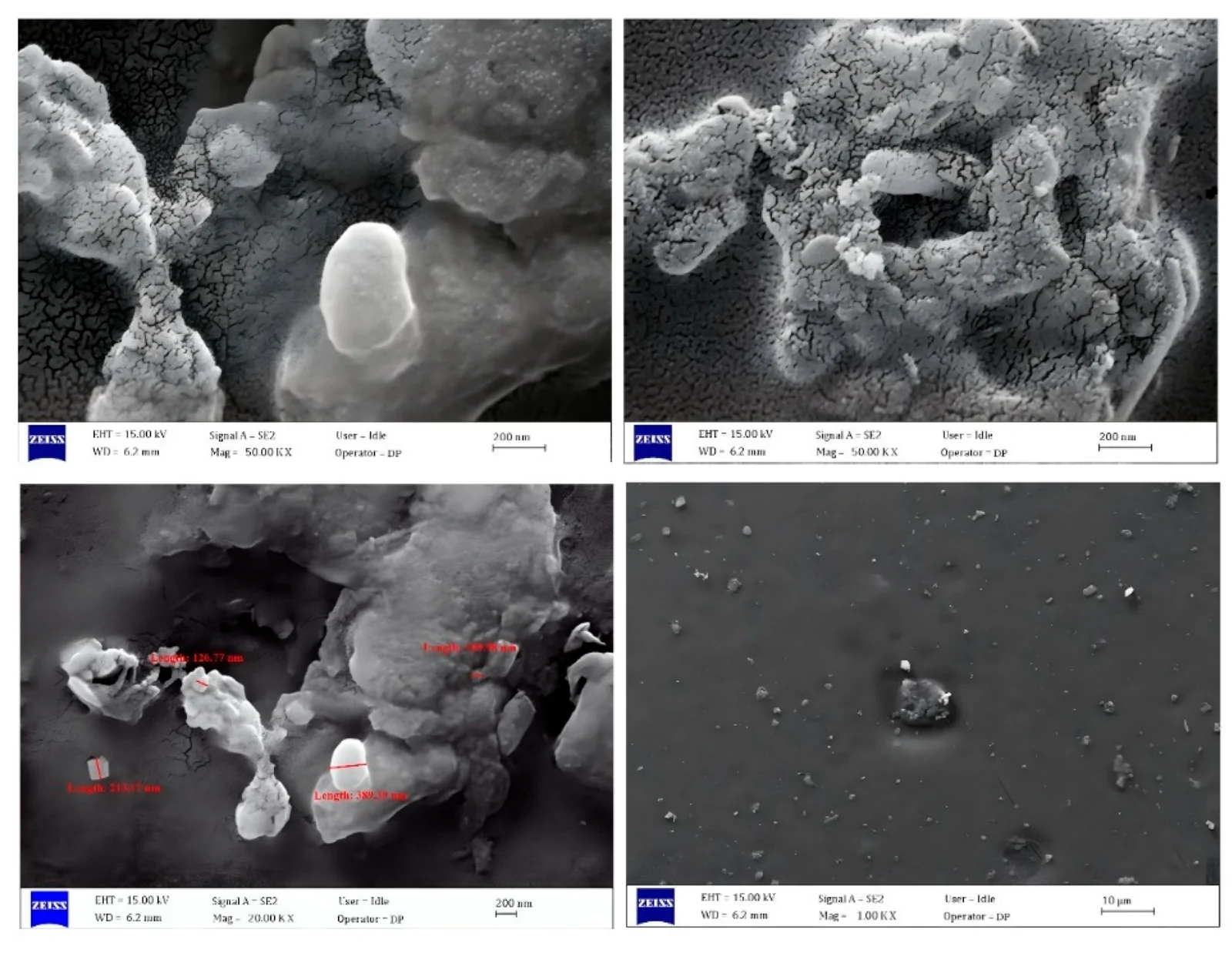
FESEM images for PVC films without nanomaterials after irradiation by UV.

**Figure 24:**
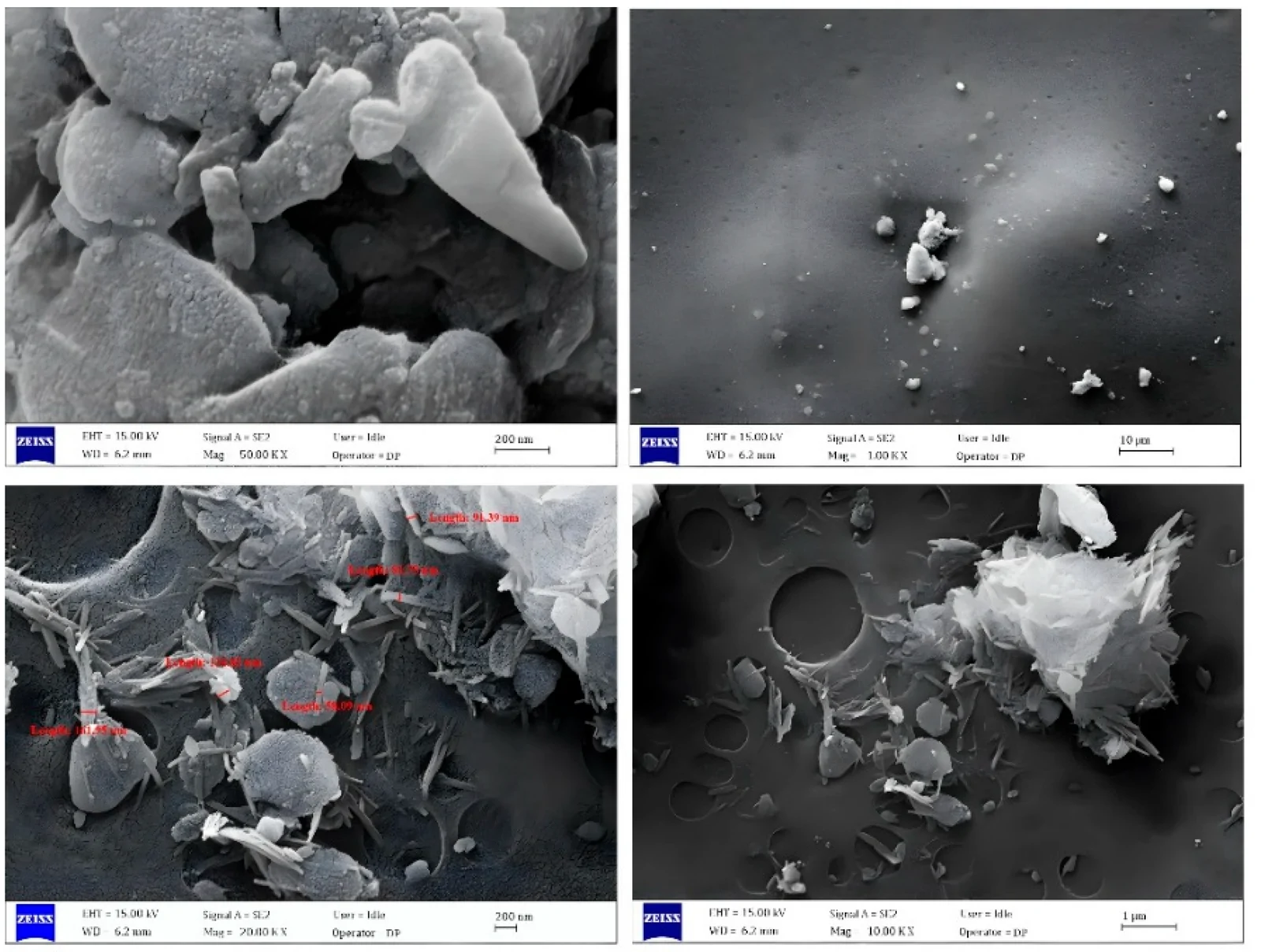
FESEM images for PS.

**Figure 25:**
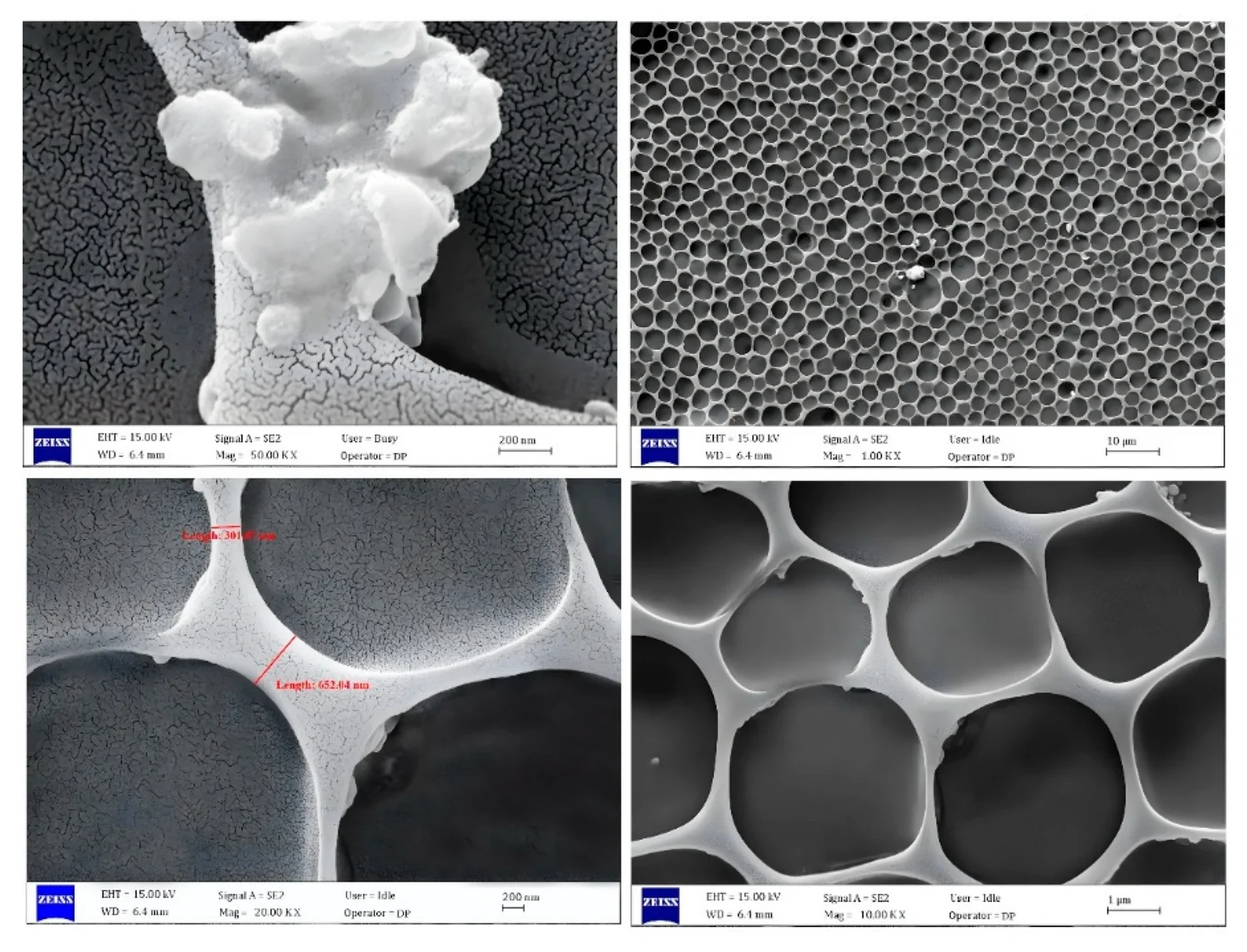
FESEM images for PSN1.

**Figure 26:**
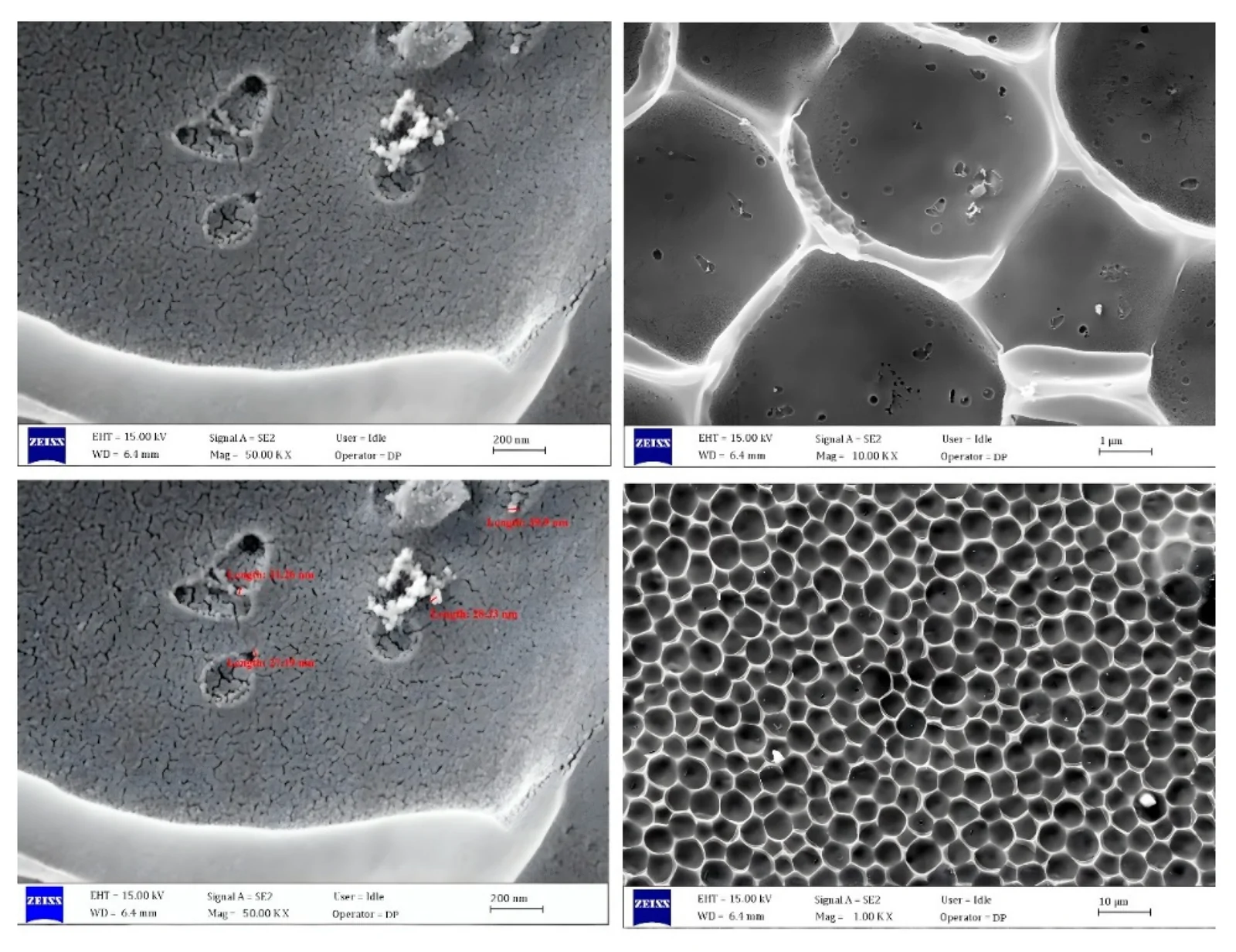
FESEM images for PSN11.

**Figure 27:**
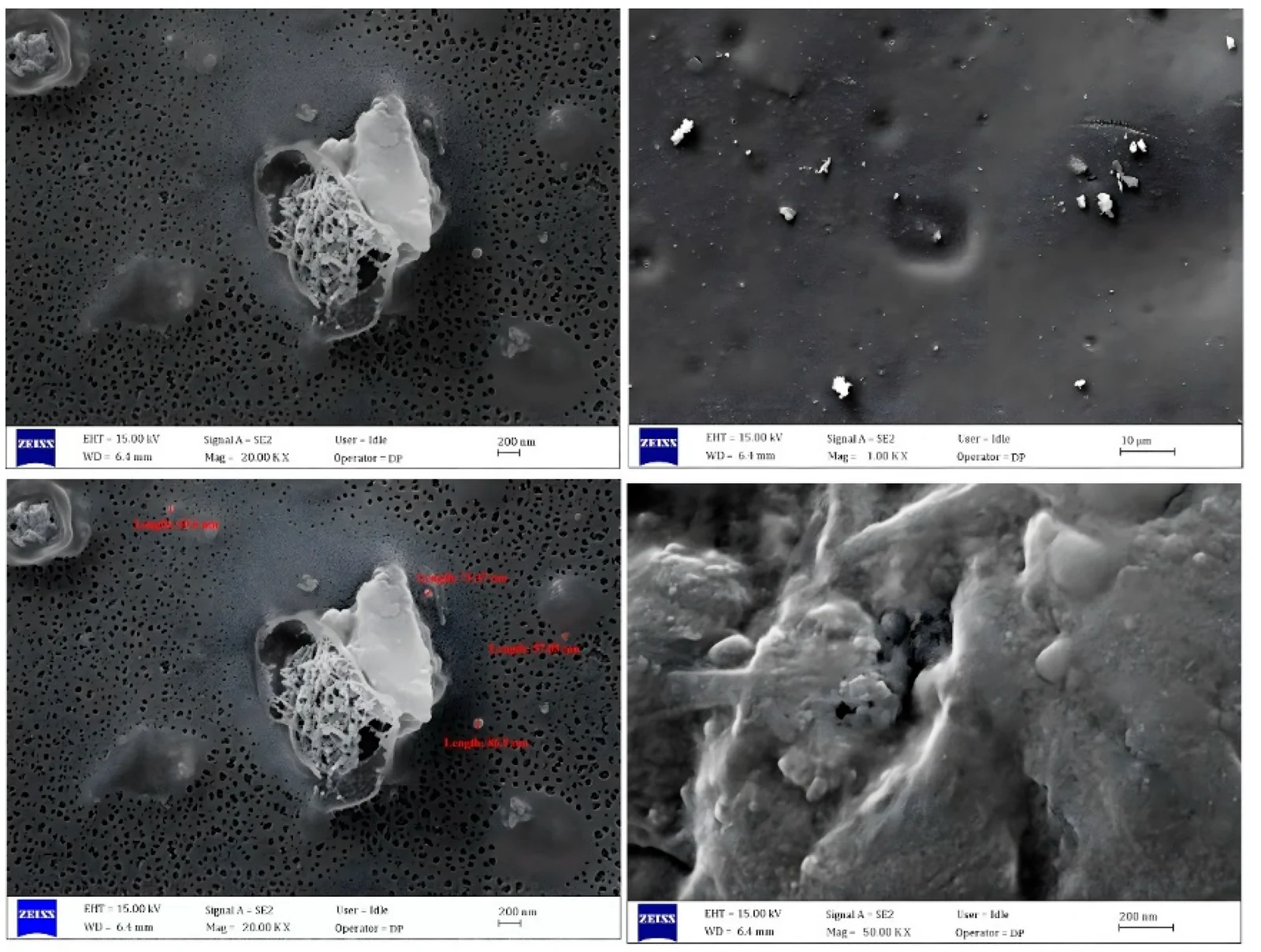
FESEM images for PSN2.

**Figure 28:**
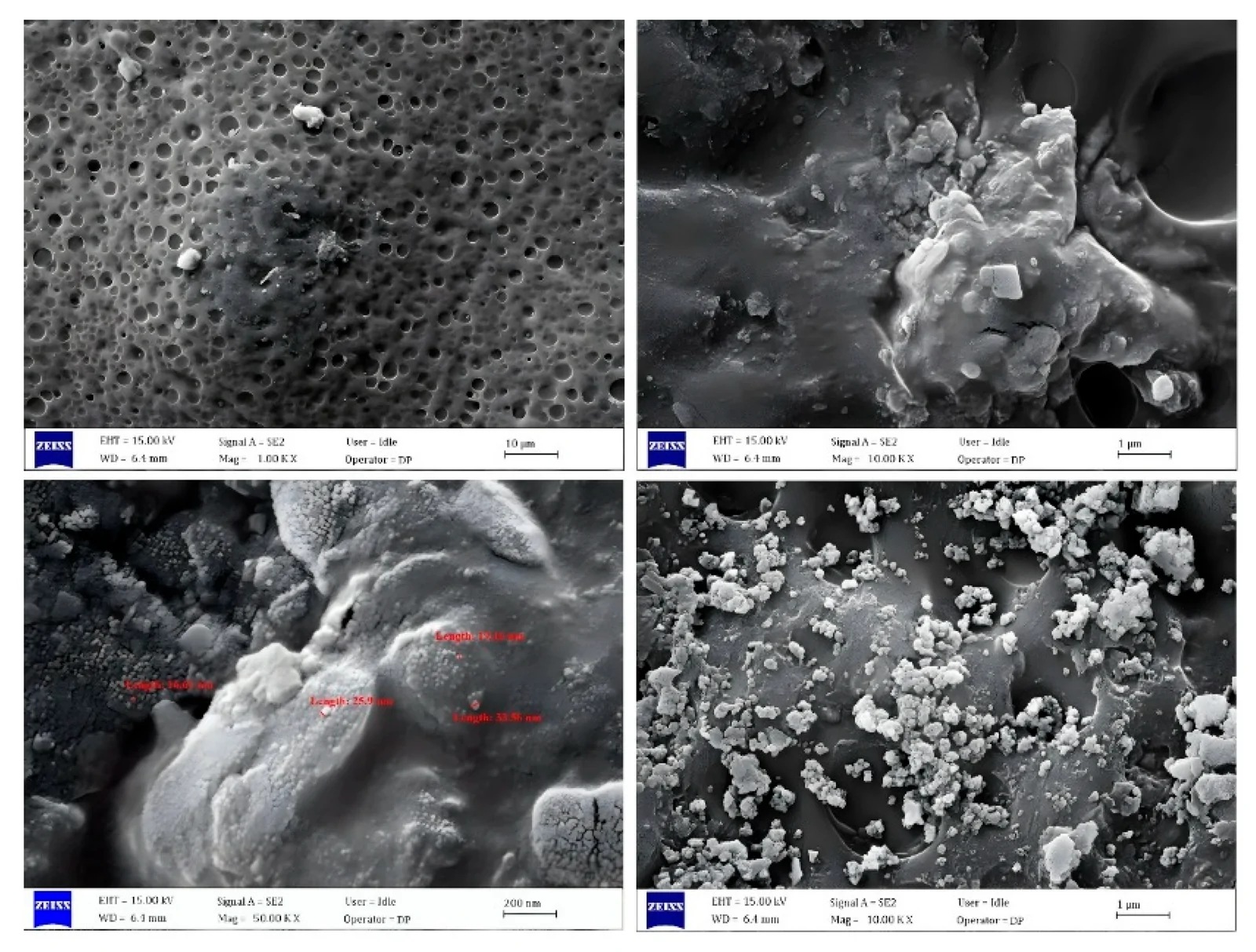
FESEM images for PSN22.

FESEM was conducted to compare the effect of UV on the surface morphology of PVC films before and after (300 h) irradiation, and the surface of the PVC film (blank) is essentially smooth and clear before the irradiation. After the irradiation for (300 h), composite films of both PVC (blank) and PVC with the photostabilizers have obviously deteriorated, and cavities with fractures appear to have formed on the surface. It can be seen that the cavities have increased in both length and depth compared to the results for the non-radiated PVC film (blank), indicating the damaging effects of irradiation on the surface of PVC films, as shown in Figure 23, Figure 24, Figure 25, Figure 26, Figure 27 and Figure 28.

FESEM results qualitatively confirmed FTIR and weight-loss data, with nanoparticle-containing films exhibiting less surface degradation than that for blank PVC. Due to varying magnifications, FESEM images were interpreted intuitively rather than quantitatively to compare pore or fracture diameters.

**Figure 29:**
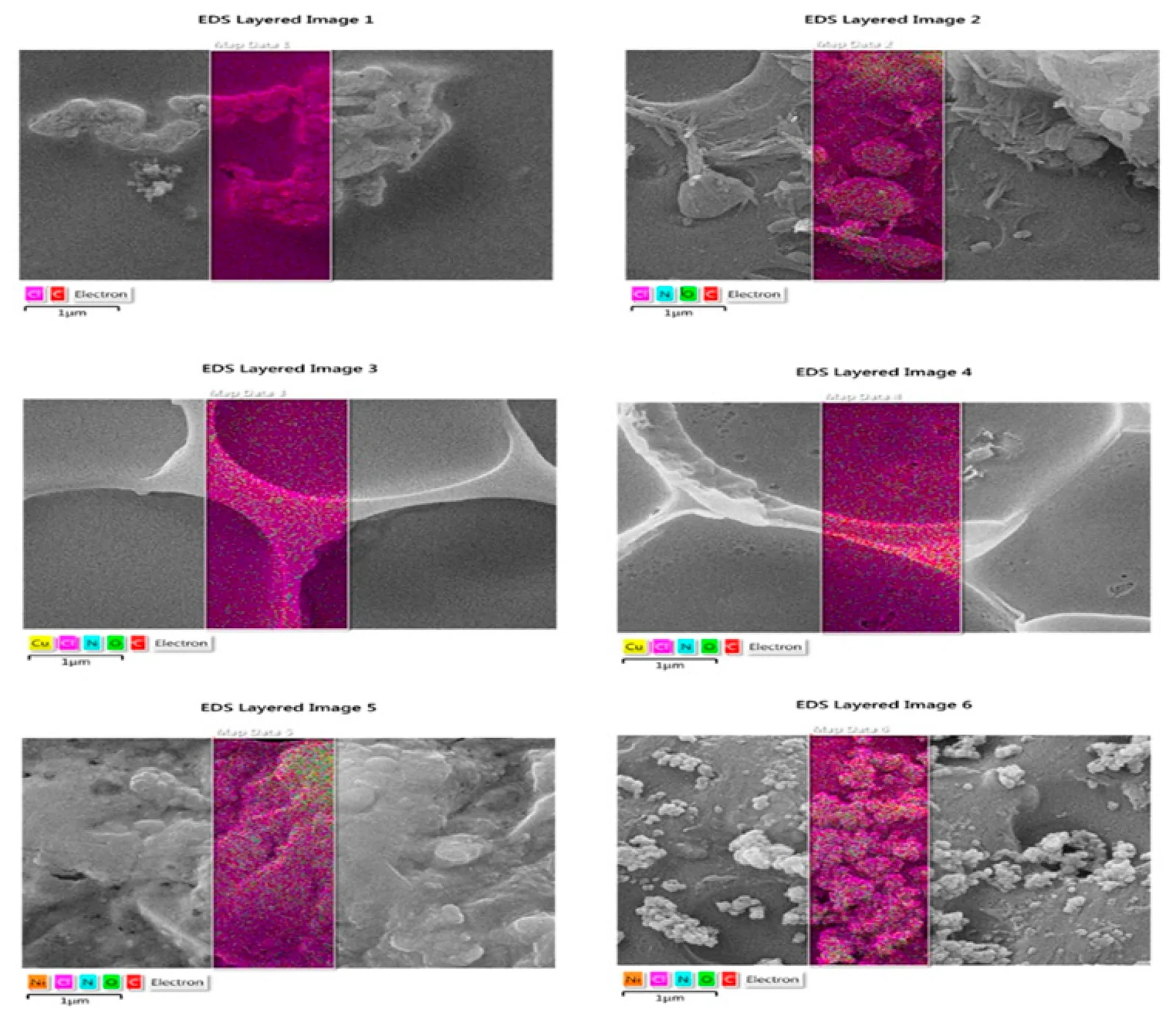
X-ray elemental mapping of UV-irradiated blank PVC and nanocomposite films after 300 h.

X-ray mapping is related to scanning electron microscopy (SEM) and is used for producing images related to the distributions and relative abundance of elements in different colors of the sample [19]. The distribution of elements and the abundance of nanomaterials on PVC surfaces decrease the photo degradation, as shown in Figure 29. After PVC exposure to UV light, dehydrochlorination occurs, and the abundance of the chlorine element decreased compared to the results for nanomaterials with PVC.

**Figure 30:**
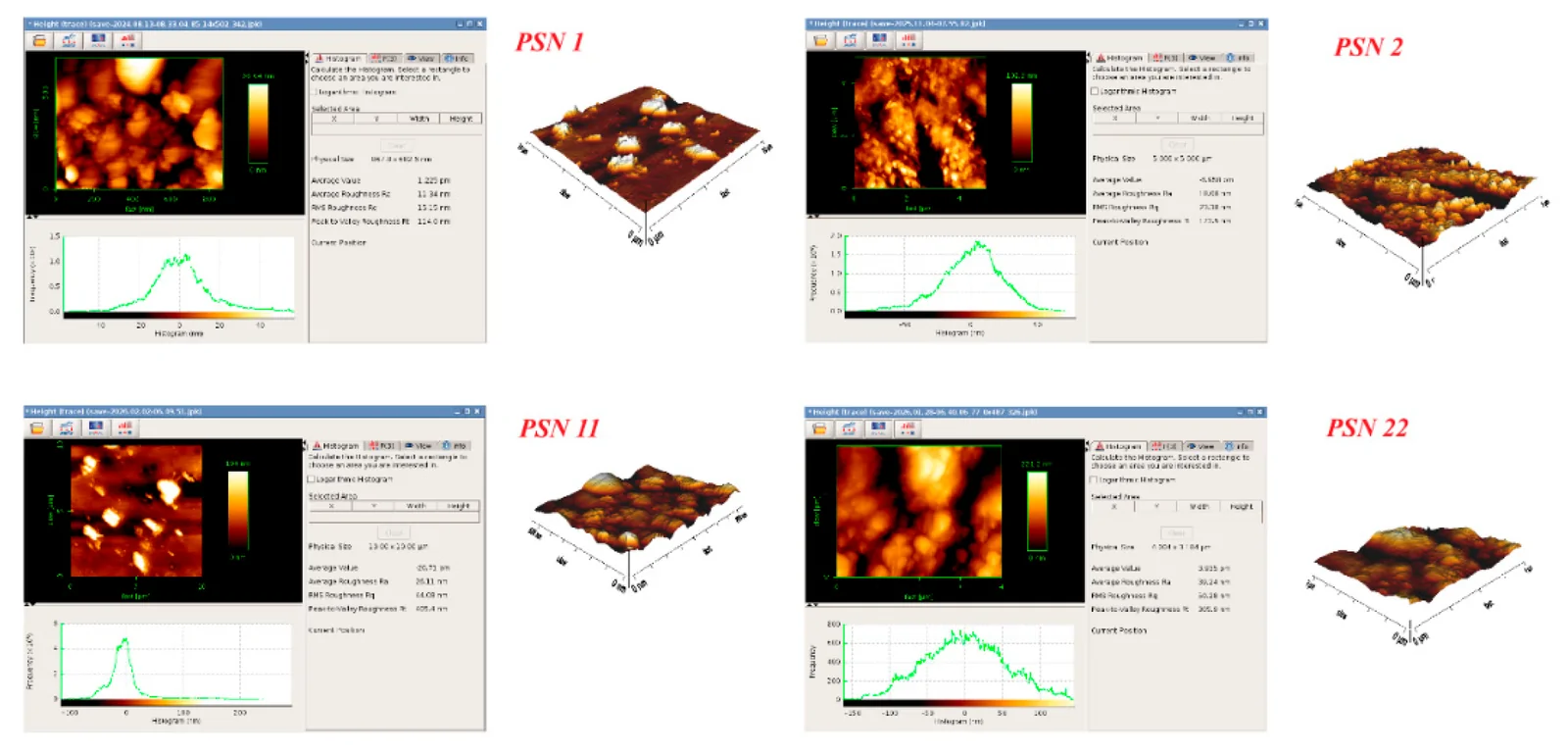
AFM images of PVC with additions after radiation.

AFM scans indicated the surface roughness variations of UV-exposed films. The PSN1 film had the smoothest surface, lowest average roughness (Ra = 11.34 nm), and lowest RMS roughness (Rq = 15.15 nm), indicating superior nanofiller dispersion and reduced UV surface degradation, i.e., Ra = 18.08 nm for PSN2, 26.11 for PSN11, and 39.24 for PSN22, which showed the greatest roughness and most surface protrusions and aggregates. Increased roughness indicates surface flaws and particle agglomeration-induced local deterioration [20], as shown in Figure 30.

The results reveal nanoparticle composition affects PVC UV-degradation resistance and that dehydrochlorination, HCl release, and conjugated polyene bonds occur with UV radiation [21]. After reacting with polymer radicals, oxygen forms carbonyl and hydroxyl groups [22]. This mechanism explains the continued rise in all FTIR degradation indicators in our investigation, and nanoparticle-containing films deteriorated slower than did blank PVC. In contrast, PSN1 provided the greatest protection against deterioration [23], and the results are as follows: carbonyl group, 0.551; blank PVC, 1.392; polyene and hydroxyl, 0.545 and 0.591, respectively. Weight loss reduced from 2.242% to 1.131%. These findings show that surface plasmon interactions absorb and deflect UV irradiation in well-dispersed AgNPs [24]. AgNPs surfaces inhibit radical propagation, limiting chain oxidation and HCl-mediated breakdown. NiO-NPs protect through UV absorption, charge transfer, and radical trapping [25]. However, particle aggregation reduces the surface area and creates a patchwork of protection. AFM measurements showed low surface roughness, with an Ra and Rq of 11.34 and 15.15 nm, respectively, for PSN1. Therefore, nanoparticle dispersion may be as important as its chemical composition, while FTIR, weight-loss, FESEM, and AFM data suggest a protective mechanism based on UV screening, radical suppression, and decreased surface oxidation [26].

**Figure 31:**
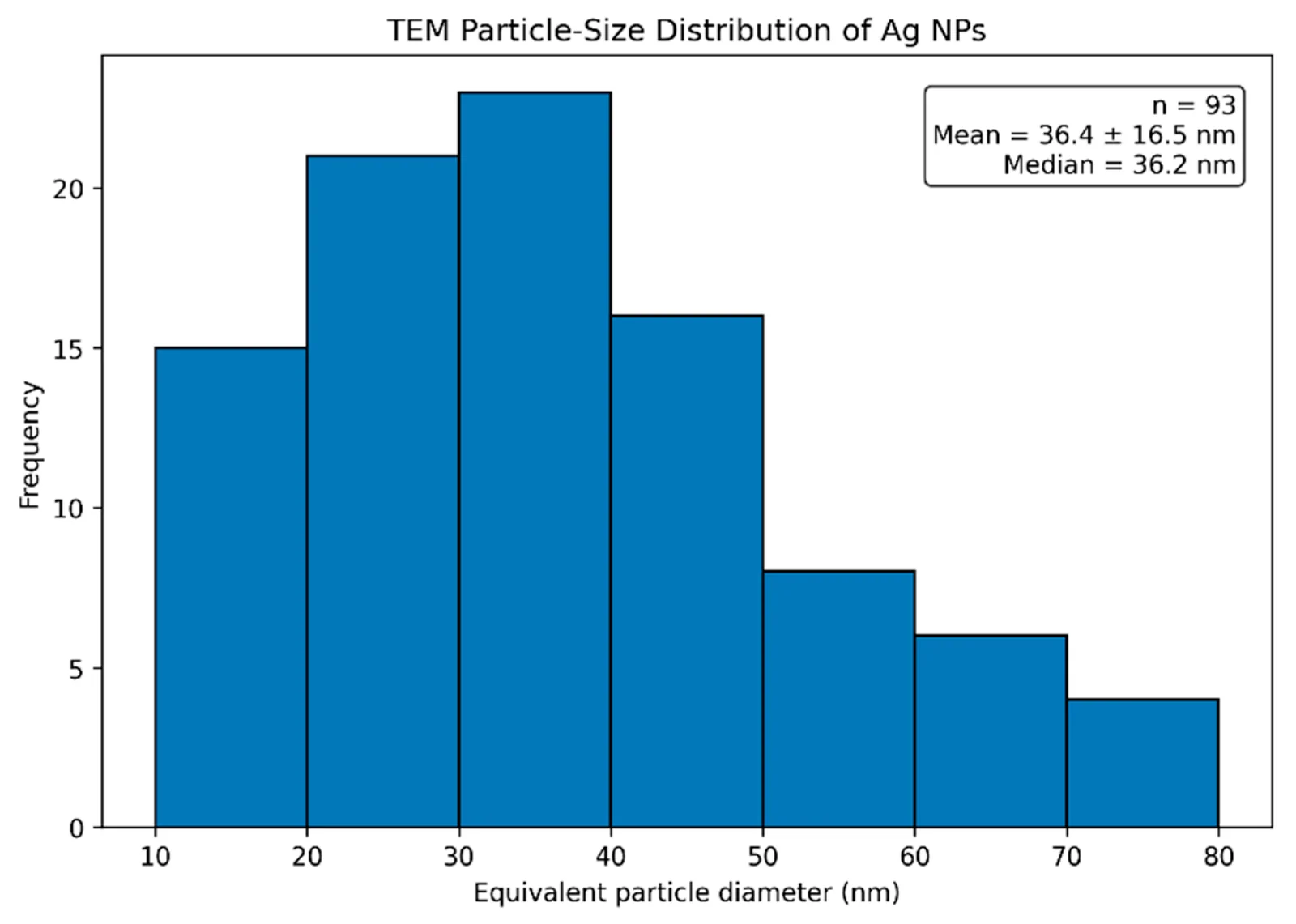
TEM micrographs and particle-size distribution of Ag NPs.

**Figure 32:**
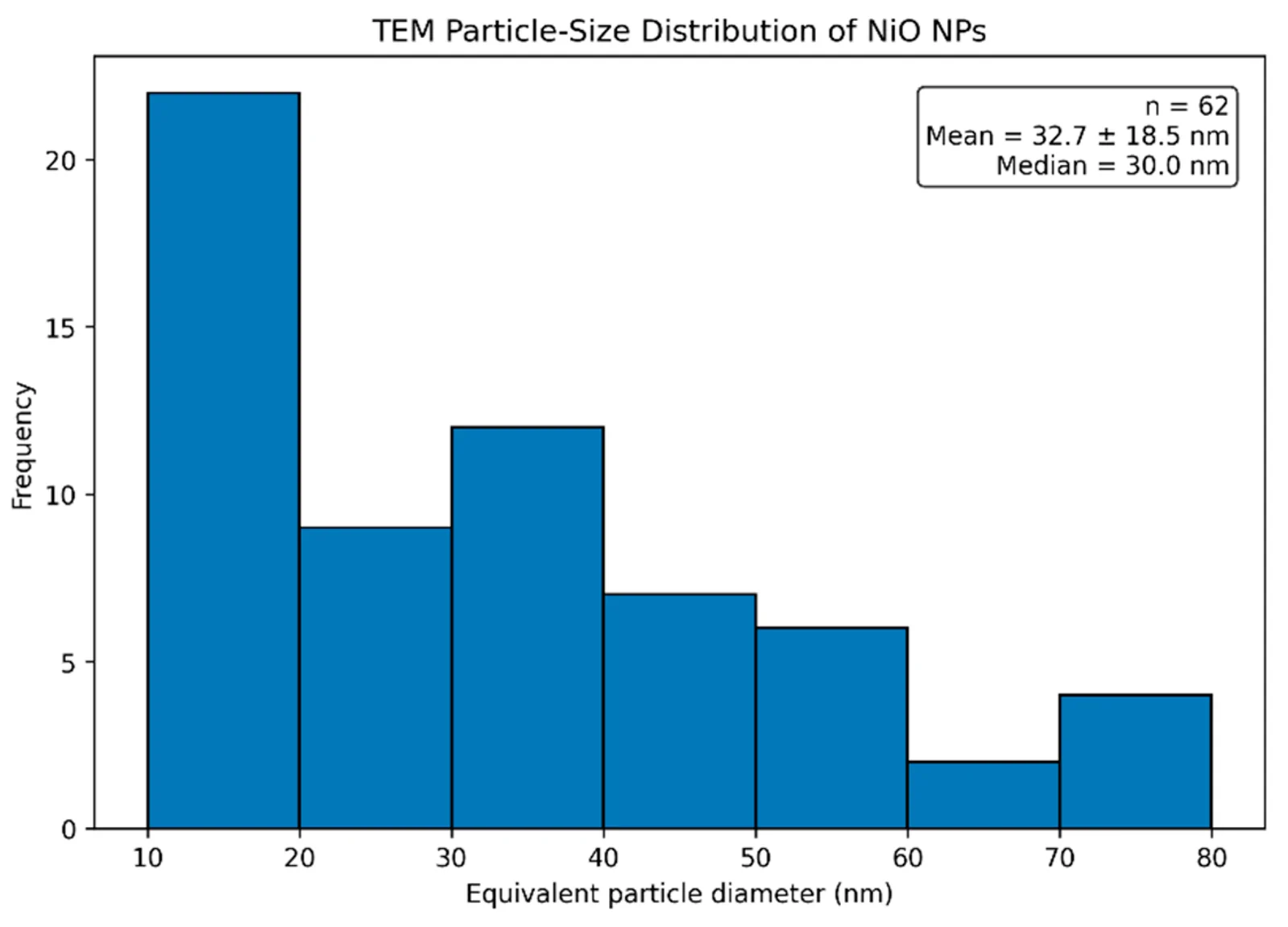
TEM micrographs and particle-size distribution of NiO NPs.

The mean equivalent diameters of the Ag and NiO nanomaterials are shown in Table 1 and Figure 31 and Figure 32.

In contrast to PVC stabilizers, Ag and NiO NPs nanoscale stabilization pathways use UV interaction, huge surface area, and radical propagation inhibition. Despite toxicity and legal restrictions, lead and organotin stabilizers are, nevertheless, effective. Safety requires regular co-stabilizers in Ca/Zn systems. UV absorbers and hindered amine stabilizers may move or degrade over time. Ag and NiO NPs exhibit aggregation, concentration-dependent performance, cost, migration and long-term toxicity issues that require exploration. The present nanomaterials are offered as potential photostabilizers, not as general replacements for PVC stabilizer systems, as shown in Table 2.

The Ag- and NiO nanomaterials containing films are protected by UV screening, interfacial interactions, and radical-mediated oxidation suppression. Although direct radical or charge-transfer measurements were not conducted in this investigation, deterioration patterns and literature studies suggest these processes.

A 0.5 wt%, compared to 0.3 wt%, photostabilization loading falls within the range studied, but a broader range of concentrations will be necessary to identify the genuine optimal loading. Future investigations should independently assess PVC polymerization following purification.

## 4. Conclusions

Green-synthesized Ag and NiO nanoparticles reduce UV-induced PVC film deterioration, with 0.3 wt% Ag NPs providing the most protection, as observed for all indices. Plant-based synthesis uses no harsh reduction agents, so industry may produce durable, weather-resistant plastics without the addition of harmful stabilizers. If the optimized ratios of Ag and NiO nanoparticles are combined, stabilization performance should be superior to the results using either alone. Future formulations should test hybrid loading with longer than 350 h of exposure to validate long-term performance. Additional investigations will include mechanical testing and outdoor field trials. Since the current findings are largely based on the results for blank film, the replication of statistics over several batches of data would increase confidence that the reported percentages can be accurately approximated, and an additive-free control group would strengthen the present study. Quantitative surface roughness data, such as AFM measurements for FESEM pictures, would also assist in determining how nanoparticle type affects surface stability after long-term irradiation. This study establishes a strong basis for low-toxicity PVC stabilization, and expanding the dataset to its conclusions will boost its practicality.

## Figures and Tables

**Table 1:** TEM particle-size characteristics of the Ag NPs and NiO NPs.

Material	n	Mean Equivalent Diameter	SD	Median
Ag NPs	93	36.35 nm	16.53 nm	36.20 nm
NiO NPs	62	32.73 nm	18.52 nm	29.98 nm

**Table 2:** Different stabilizers and their main advantages and limitations.

Stabilizer	Main Advantage	Main Limitation
Lead salts	Strong thermal stabilization	Toxicity and environmental restrictions
Organotin compounds	High stabilization efficiency	Toxicity, cost, regulatory limitations
Ca/Zn systems	Lower toxicity	Often require co-stabilizers
UV absorbers	Direct UV screening	Migration and gradual loss of activity
HALS	Radical-scavenging activity	Limited performance under some PVC conditions and possible additive interactions
Ag NPs	UV absorption/scattering, radical suppression, high surface area	Cost, aggregation, nanoparticle safety require further evaluation
NiO NPs	UV absorption, semiconductor behavior, radical-trapping contribution	Aggregation, dispersion dependence, nanoparticle safety require further evaluation

## Data Availability

The original contributions presented in this study are included in the article. Further inquiries can be directed to the corresponding authors.
